# The Role of Membrane Affinity and Binding Modes in Alpha-Synuclein Regulation of Vesicle Release and Trafficking

**DOI:** 10.3390/biom12121816

**Published:** 2022-12-05

**Authors:** Tapojyoti Das, Meraj Ramezani, David Snead, Cristian Follmer, Peter Chung, Ka Yee Lee, David A. Holowka, Barbara A. Baird, David Eliezer

**Affiliations:** 1 Department of Biochemistry, Weill Cornell Medical College, New York, NY 10065, USA; 2 Department of Structural Biology, St. Jude Children’s Research Hospital, Memphis, TN 38105, USA; 3 Department of Chemistry and Chemical Biology, Cornell University, Ithaca, NY 14853, USA; 4 Metabolism Program, The Broad Institute of MIT & Harvard, Cambridge, MA 02142, USA; 5 Department of Biochemistry and Molecular Biology, Johns Hopkins University, Baltimore, PA 21205, USA; 6 Laboratory of Biological Chemistry of Neurodegenerative Disorders, Department of Physical-Chemistry, Institute of Chemistry, Federal University of Rio de Janeiro, Rio de Janeiro 22290-240, Brazil; 7 Department of Chemistry, James Franck Institute, Institute of Biophysical Dynamics, The University of Chicago, Chicago, IL 60637, USA; 8 Department of Physics and Astronomy and Department of Chemistry, University of Southern California, Los Angeles, CA 90089, USA

**Keywords:** alpha-synuclein, membrane, synaptic vesicle, synapsin, Parkinson’s

## Abstract

Alpha-synuclein is a presynaptic protein linked to Parkinson’s disease with a poorly characterized physiological role in regulating the synaptic vesicle cycle. Using RBL-2H3 cells as a model system, we earlier reported that wild-type alpha-synuclein can act as both an inhibitor and a potentiator of stimulated exocytosis in a concentration-dependent manner. The inhibitory function is constitutive and depends on membrane binding by the helix-2 region of the lipid-binding domain, while potentiation becomes apparent only at high concentrations. Using structural and functional characterization of conformationally selective mutants via a combination of spectroscopic and cellular assays, we show here that binding affinity for isolated vesicles similar in size to synaptic vesicles is a primary determinant of alpha-synuclein-mediated potentiation of vesicle release. Inhibition of release is sensitive to changes in the region linking the helix-1 and helix-2 regions of the N-terminal lipid-binding domain and may require some degree of coupling between these regions. Potentiation of release likely occurs as a result of alpha-synuclein interactions with undocked vesicles isolated away from the active zone in internal pools. Consistent with this, we observe that alpha-synuclein can disperse vesicles from in vitro clusters organized by condensates of the presynaptic protein synapsin-1.

## 1. Introduction

Alpha-synuclein is a 140-amino-acid protein genetically and pathologically linked to Parkinson’s disease (PD). First described by James Parkinson in 1817, PD is a progressive neurodegenerative disorder that affects more than 6 million people globally [1], with an increasing prevalence in the aging population. The disease is characterized by a triad of clinical symptoms—resting tremor, bradykinesia and rigidity—resulting from a progressive loss of dopaminergic neurons in the substantia nigra in the basal ganglia of the midbrain. Although the disease was clinically described long ago, a causal link between alpha-synuclein and PD was only established 180 years later [2].

In PD, alpha-synuclein is a major constituent of characteristic intraneuronal deposits known as Lewy bodies and Lewy neurites in the form of highly ordered amyloid fibril aggregates [3]. Despite great efforts to clarify the relation of these aggregates and their formation to PD, their role in the etiology of PD remains poorly understood. Relatively less effort has been expended to understand the normal physiological functions of alpha-synuclein because the relevance of such functions to disease are unclear. Nevertheless, based on a variety of observations in different contexts, a consensus has emerged that alpha-synuclein is a modulator of synaptic function. Before being linked to PD, alpha-synuclein was described as a novel protein enriched in synaptic vesicle (SV) preparations in the giant neurons of the electric ray *Torpedo californica* [4]. Early studies in songbirds suggested a role in song learning [5], while studies of knockout (KO) models have reported diverse effects on postsynaptic potentiation [6], paired pulse and frequency facilitation [7], SV pool size [8], 4-aminopyridine responses [9], increased neurotransmitter release [10,11,12] and other more subtle impairments [13,14]. The relatively few studies that examined KO of all three synuclein family members (alpha-, beta- and gamma) consistently showed an increase in synaptic transmission [12,15], suggesting an inhibitory role for the synucleins. Overexpression of alpha-synuclein in various contexts has also generally been reported to lead to a decrease in neurotransmitter release [7,16,17,18,19,20], although a few studies have instead reported increased release [6,13].

SV release is a highly regulated process culminating in the fusion of docked vesicles with the synaptic plasma membrane, a process that is itself regulated by a number of proteins and protein complexes [21]. SVs are organized in physiological pools defined by shared functional properties [22,23] and differing in their ability to release in response to a stimulus. The classical three-pool model comprises a readily releasable pool that is immediately released on stimulation, a recycling pool that is released upon moderate stimulation and a reserve pool that is only mobilized and released upon intense or repetitive stimulation of the neuron [22]. Synucleins have been reported to alter SV pool sizes in functional studies [16] as well as to affect vesicle clustering as observed in ultrastructural studies [24].

Structurally, alpha-synuclein has been shown to be an intrinsically disordered protein, lacking secondary and tertiary structure when isolated in solution. In the presence of membranes, its lipid-binding domain (residues 1–94) acquires an alpha-helical structure, which can take the form of a single extended helix spanning the entire lipid-binding domain or of two shorter helices (helix-1 spanning residues 1–37 and helix-2, spanning residues 45–94) linked by a non-helical and somewhat flexible linker. While membrane binding of alpha-synuclein has long been assumed to be important for its regulation of SV trafficking and fusion, a mechanistic understanding of the underlying structure–function relationships has remained elusive. Recently, we developed a non-neuronal model system for alpha-synuclein function using assays of calcium-triggered exocytosis of recycling vesicles in RBL-2H3 cells, which are used as a model for immune system secretory cells. We found that alpha-synuclein functions as both an inhibitor and a potentiator of vesicle release in a concentration-dependent manner, within the range of reported physiological concentrations. Using structure–function studies of specific mutants, we suggested that different lipid-binding modes are associated with inhibition and potentiation, with the broken-helix conformation being critical for inhibitory function and the extended-helix state mediating potentiation of vesicle release. We also found that potentiation of release at high alpha-synuclein concentrations is associated with dispersal of vesicles from the endocytic recycling compartment, a membranous organelle typically localized in the perinuclear region, to the cell periphery.

Despite these novel insights, critical questions remain regarding the link between specific membrane-bound conformations and alpha-synuclein function. Given the proposed roles of the broken- and extended-helix states, we noted that the linker region between helix-1 and helix-2 (residues 38–44) is ideally situated to regulate alpha-synuclein conformation and function. Indeed, we previously reported that phosphorylation of Y39 within the linker region by c-Abl kinase alters the lipid-binding conformation of alpha-synuclein [25]. Interestingly, dysregulation of c-Abl activity in phosphorylation alpha-synuclein at residue Y39 has been linked to PD [26,27,28], providing a potential link between the physiological functions and pathological roles of the protein. To probe the role of the linker region and of the broken- and extended-helix states of alpha-synuclein in the regulation of exocytosis, we report here studies of two conformationally selective mutants of alpha-synuclein, correlating their effects on alpha-synuclein structure when bound to membranes and membrane mimetics with their functional effects in assays of vesicle release in RBL-2H3 cells. We find that stabilizing the extended-helix state of alpha-synuclein enhances potentiation of release. More generally, this activity correlates with binding affinity for isolated vesicles. Surprisingly, promoting the broken-helix state via helix-perturbing mutations in the linker region reduced inhibition of vesicle release, indicating that additional factors beyond two flexibly coupled helices contribute to this activity of the protein.

## 2. Methods

### 2.1. Expression and Purification of Alpha-Synuclein Variants

The wild-type human alpha-synuclein sequence, cloned into a pT7.7 vector, a kind gift from Peter Lansbury [29,30], was used as the starting point for all alpha-synuclein expression constructs. Site-directed mutagenesis was performed using QuikChange site-directed mutagenesis kit (Agilent), and all resulting sequences were confirmed by DNA sequencing. To express N-terminal acetylated proteins in *E. coli* BL21(DE3) cells, the alpha-synuclein plasmid was co-transformed with a pNatB plasmid, containing the yeast *N*-acetyltransferase complex NatB, a kind gift from Prof. Daniel Mulvihill (University of Kent, Canterbury, UK) via Prof. Elizabeth Rhoades (University of Pennsylvania, Philadelphia, PA, USA) [31,32]. Transformed BL21(DE3) cells were grown in either LB media (for unlabeled proteins) or M9 minimal media supplemented with ^15^N-NH_4_Cl and either unlabeled or ^13^C-labeled glucose as the sole nitrogen and carbon source to produce ^15^N- or ^15^N^13^C-labeled recombinant proteins for NMR experiments. Protein expression was induced by addition of IPTG to 0.84 mM at 0.6 OD, and optical density was monitored until a peak was reached (1.2–1.5 OD) about 2–3 h later. Cultures were harvested by centrifugation at 10,000× *g* for 15 min at 4 °C, and the pellet was stored frozen at −20 °C or below until purification.

Thawed bacterial pellets were resuspended in 50 mL of lysis buffer (20 mM Tris-Cl at pH 8.0, 1 mM EDTA, 1 mM PMSF) by vortexing, sonicated on wet ice for a total of 12 min (2 times 6 min with stirring in between) and ultracentrifuged for 45 min at 200,000× *g* (40,000 rpm on a Beckman 50.2 Ti rotor), and the supernatant was collected. The pH of the ultracentrifuge supernatant was lowered to 3.5 using 1M HCl and centrifuged at 40,000× *g* for 15 min at 4 °C to remove contaminating proteins, as previously described [33,34]. The pH was readjusted to 7.5 using 1M NaOH, and alpha-synuclein was precipitated by salting-out method using ammonium sulfate at 50% saturation (0.291 g/mL). The precipitate was collected by centrifugation at 40,000× *g* for 15 min at 4 °C, dissolved in 25 mL Tris-Cl buffer (pH 8) and dialyzed through a 3.5 kDa cutoff membrane against water, changing the dialysate twice. After dialysis, the protein was flash-frozen in liquid nitrogen and lyophilized to store indefinitely. A truncated version of the 3AE mutant, used for obtaining resonance assignments in the SDS-bound state, was produced by inserting a stop codon at position 102. Purification proceeded for the full-length protein until cell lysis and ultracentrifugation. Subsequently, 1% *w*/*v* streptomycin sulfate was added to the ultracentrifuge supernatant (0.5 g for 50 mL supernatant), which was stirred at 4 °C for 30 min to precipitate nucleic acids, and then centrifuged at 40,000× *g* at 4 °C for 15 min. The supernatant was subjected to two successive ammonium sulfate cuts, starting with addition of 0.116 g/mL, followed by centrifugation, collecting the supernatant and adding an additional 0.129 g/mL to precipitate the alpha-synuclein-containing fraction. The pellet was resuspended in 50 mL of lysis buffer (20 mM Tris-Cl at pH 8.0, 1 mM EDTA, 1 mM PMSF) and dialyzed overnight against 1L of dialysis buffer (25 mM Tris-Cl pH 8, 20 mM NaCl, 1 mM EDTA) at 4 °C. The protein solution was further subjected to cation exchange chromatography using a CM Sepharose column equilibrated with ion exchange Buffer A (25 mM Tris-Cl pH 8, 20 mM NaCl, 1 mM EDTA) before loading the sample. Sample fractions were eluted with a salt gradient from 0 to 40% ion exchange buffer B (25 mM Tris-Cl pH 8, 1 M NaCl, 1 mM EDTA) over 4 column volumes. Fractions were run on 18% SDS-PAGE to determine those containing the purified protein, which were pooled, dialyzed against 5% acetic acid overnight and passed through a 0.22 µM membrane filter to remove particulate impurities. The sample was then injected onto a reversed phase C4 column using a Waters 2690 separation module and eluted with a gradient of 20% to 100% HPLC Buffer B (90% acetonitrile and 0.1% trifluoroacetic acid in water) in HPLC Buffer A (0.1% trifluoroacetic acid in water) while monitoring the eluate for protein absorbance at 229 nm. Major peaks from HPLC were collected separately and analyzed by SDS-PAGE. Protein-containing fractions were spun in a vacuum concentrator for 2 h to remove acetonitrile, dialyzed in water to remove residual TFA, flash frozen in liquid nitrogen and lyophilized for storage.

### 2.2. Lipid Vesicle Preparation and Characterization

Synthetic lipids were purchased as stock solutions in chloroform (Avanti Polar Lipids, Alabaster, AL, USA), and appropriate volumes were mixed at a molar ratio of DOPC/DOPE/DOPS = 60:25:15 to mimic the composition of native SVs [35,36,37]. The lipids were dried under nitrogen or argon flow for 20 min while rolling the tube to form a thin film on the side, followed by drying in a vacuum concentrator for a further 2 h. If not immediately needed, the tube was flushed with nitrogen or argon, sealed with paraffin film and stored at −20 °C for up to 3 days. The lipid film was hydrated in an appropriate volume of NMR buffer (10 mM Na_2_HPO_4_, 100 mM NaCl, 10% D_2_O, pH 6.8) and vortexed vigorously to generate a cloudy suspension, which was sonicated using a bath sonicator (Elmasonic P30H) at room temperature, 37 kHz frequency and 100 W power for 20–30 min until visually clear. Clarity of the preparation ensures that the vesicles are sufficiently small (<100 nm diameter) to become non-scattering to visible light. Next, the vesicle suspension was ultracentrifuged at 150,000× *g* (60,000 rpm on a Sorvall S120-AT2 rotor) to pellet larger particles. The supernatant was carefully removed and collected in a fresh tube and used within 2 days to prepare NMR samples by adding to protein stock solution at specific lipid/protein ratios. In order to determine concentration of lipids in vesicles, we performed a phosphate assay based on a modified version of the Rouser assay for phospholipids [38,39]. The size distribution of vesicles was measured for select SUV preparations using dynamic light scattering, which showed that the vesicle preparation method consistently produces vesicles of 30–50 nm diameter.

### 2.3. NMR

To prepare samples for NMR, lyophilized proteins were weighed out and dissolved in NMR buffer (10 mM Na_2_HPO_4_, 100 mM NaCl, 10% D_2_O, pH 6.8), pH readjusted to 6.8 and filtered using a 100 kDa cutoff centrifugal filter (MilliporeSigma, Burlington, MA, USA) to remove any higher molecular weight species/aggregates. Protein concentration was estimated from absorbance at 280 nm using a molar extinction coefficient of 5120 M^−1^cm^−1^ [30]. NMR experiments were conducted using Bruker 600 MHz, 700 MHz, 800 MHz and 900 MHz NMR instruments with triple resonance gradient-equipped cryoprobes. Pulse sequences, except for the DEST experiments, were derived from the standard Bruker library. NMR tubes were either 5 mM thin-walled precision tubes (Wilmad-LabGlass, Vineland, NJ, USA) or 3 mM Bruker SampleJet NMR tubes (Bruker, Billerica, MA, USA). Experiments were conducted at 10 °C for free-state and lipid vesicle-binding experiments and at 40 °C for samples containing SDS. For relaxation experiments, the temperature was set at 13.5 °C because the temperature control of one of the spectrometers used was better at this temperature. NMR raw data conversion and processing were conducted using NMRPipe [40], and data analysis and visualization were performed using CCPNmr Analysis [41].

^15^N R_2_ relaxation rates were measured by conducting a series of HSQC-like experiments with variable time delays when the amide nitrogen magnetization is transverse, with a number of Carr–Purcell–Meiboom–Gill (CPMG) refocusing elements interspersed. Each CPMG delay–pulse–delay element duration was 16.32 ms, and variable numbers of such pulses (0 to 18) corresponded to a maximum of 294 ms relaxation delay. The multiplier array for the number of CPMG pulses was scrambled, and the delay between each complete pulse sequence was set at 4 s to prevent sample overheating. A single data point in the mid-range relaxation delay was chosen and measured in triplicate to obtain an error estimate. The signal intensity for each amide peak was plotted as a function of the time delay and fit to a single exponential to extract the R_2_ relaxation rate.

Resonances of vesicle-bound residues are broadened beyond direct detection, but this broad resonance can be selectively saturated using a narrow bandwidth saturation pulse far away from the corresponding free-state resonance frequency. When this saturated bound state exchanges with the free state, the signal from the free state decreases consequently. This technique is referred to as Dark-state Exchange Saturation Transfer (DEST) and, in combination with R_2_ relaxation experiments, is a powerful method to probe the kinetics of exchange processes occurring at timescales ranging from approximately 10 ms to 1 s [42]. Using these experiments, it is possible to uncover which residues are interacting with the membrane, to evaluate kinetic models for the system and to extract the kinetic rates of membrane interactions.

DEST and R_2_ relaxation experiments were performed on either a 700 MHz or a 900 MHz spectrometer based on instrument availability, with data from a single spectrometer used for each individual sample. DEST experiments were performed using a saturation pulse of 900 ms on the ^15^N channel at resonance offsets ranging from −30 kHz to +30 kHz, using two different saturation bandwidths (400 Hz and 175 Hz on the 700 MHz spectrometer and 500 Hz and 200 Hz on the 900 MHz spectrometer). For each bandwidth, the saturation pulse power was calculated from the ^15^N 90° pulse length assuming an ideal linear amplifier.

For both R_2_ relaxation and DEST experiments, peak picking, annotation and height measurements and (for R_2_ relaxation) exponential decay modeling were performed using the NMRPipe suite using scripts originally developed by Fawzi et al. [42] and subsequently modified by us to accept different input formats. Joint fitting of R_2_ relaxation and DEST profiles to the different kinetic models was carried out using the DESTfit MATLAB script [42], which essentially fits the experimental values to a homogenous form of the McConnell equations, describing a single spin in two-site exchange at chemical equilibrium between a free state with low R_2_ and an SUV-bound state with larger R_2_ in the presence of a continuous-wave saturation field [43,44]. The models tested include a simple two-state model for membrane binding where the protein exchanges between a free state and a fully bound state and the overall exchange process are the same for each residue (a single global apparent on-rate, *k_on_^app^*, and a single global off-rate, *k_off_*) and a pseudo-two-state model, as described by Fawzi et al. [42], where bound-state conformations are divided in two subsets, with the *i^th^* residue either in direct contact with the vesicle surface or tethered to the vesicle surface by other nearby residues that are in direct contact (Figure S10). The global apparent on-rate, *k_on_^app^(i)*, is then the sum of the apparent on-rates for binding in the tethered contact mode, *k_1_^app^(i)*, and in the direct contact mode, *k_2_^app^(i)*, and the global off-rate, *k_off_ = k_−1_* = *k_−2_* is considered the same for these two states for all residues. The residue-specific equilibrium between the tethered and direct contact states, described by *K_3_(i)* = *k_2_^app^(i)*/*k_1_^app^(i),* then relates the populations of the tethered contact and direct contact states. The pseudo-two-state model can also incorporate direct interconversion between the tethered and the direct contact states with rate constants *k_3_* and *k_−3_*, but this interconversion only becomes relevant if the rates involved are faster than the off-rate *k_off_*. In our case, addition of *k_3_* and *k_−3_* to the fitting parameters did not improve the fits for any of the variants, and the resulting values were lower than *k_off_*, indicating that interconversion is relatively slow and is not contributing meaningfully to our measurements.

### 2.4. Paramagnetic Relaxation Enhancement Experiments

To incorporate a nitroxide spin label at a single-cysteine mutant of alpha-synuclein, ^15^N-labeled alpha-synuclein was dissolved in 1mL of PRE buffer (10 mM Na_2_HPO_4_, 100 mM NaCl, pH 6.8) at a concentration of 200–400 µM. Then, the spin label reagent MTSL (S-(1-oxyl-2,2,5,5-tetramethyl-2,5-dihydro-1H-pyrrol-3-yl)methyl methanesulfonothioate) was added at a 30-fold molar excess (from a 300 mM stock solution in acetonitrile) and allowed to react for a couple of hours at room temperature. Next, the unbound spin label was removed by dialyzing against 1 L of PRE buffer through a 3.5 kDa cutoff membrane, with one change of dialysate. Finally, 10% *v*/*v* D_2_O was added to prepare the sample for NMR experiments. A matched control sample was also prepared by adding DTT to a final concentration of 5 mM to detach the spin label by reducing the disulfide bond.

### 2.5. Tryptophan Fluorescence

For each of the alpha-synuclein variants used in the study, single tryptophan mutants at position 4 (F4W) were created by site-directed mutagenesis. Samples were prepared using 0.1 µM of protein and a series of intermediate lipid concentrations of 10, 1, 0.1 and 0.01 mM, which were further diluted in twofold steps to fill in the intervening concentration ranges. A lipid-free protein sample was also prepared for each of the variants. A protein-free lipid concentration series of samples was also prepared to measure reference spectra at each lipid concentration, which were subtracted from corresponding measurements with protein to account for absorption and scattering due to lipids alone. To assess consistency of measurements performed over multiple days using multiple batches of lipid SUV preparations, wild-type alpha-synuclein (F4W) was measured as an internal control on all the days. Fluorescence spectra were recorded using a Spectramax M5 fluorimeter (Molecular Devices, Silicon Valley, CA, USA) using a transparent quartz cuvette (Starna cells # 9F-Q-10, excitation path length 10 mM, emission path length 4 mM) using excitation at 280 nm. Emission was recorded from 300–500 nm using 10 nm steps, with the PMT potential difference set at 900 V, 60 flashes averaged.

Collecting at 10 nm resolution enabled the collection of a large data set, but the low resolution precluded a direct estimate of peak position and intensity. To determine these, we fit each fluorescence spectrum using a biparametric log-normal model that was described for organic fluorophores, including tryptophan residues in proteins (Figure S3A) [45,46]. The model, originally described by Siano and Metzler [47], postulates that fluorescence emission spectrum of single organic fluorophores empirically correspond to a log-normal distribution on the frequency axis. It is expressed as follows:

(1) $$I_{\nu }=\left\{\begin{array}{c} I_{m}\cdot \;e^{\left\{-\frac{\ln2}{\ln^{2}\;\rho }\;\cdot \;\ln^{2}\left(\frac{a-\nu }{a-\nu_{m}}\right)\right\}}\;\mathrm{at}\;\nu <a\\ 0\;\mathrm{at}\;\nu \geq a \end{array}\right.$$

where $I_{m}$ is the maximum fluorescence intensity at $\nu_{m}$, the wavenumber of the fluorescence maximum; ν is the wavenumber, which is the reciprocal of the wavelength λ; ρ is the band asymmetry parameter, which can be described as

(2) $$\rho =\frac{\nu_{m}-\nu_{-}}{\nu_{+}-\nu_{m}}$$

in which $\nu_{+}$ and $\nu_{-}$ are the wavenumber positions at the left and right half-maximal amplitudes, respectively; a is the function limiting point, described as

(3) $$a=\nu_{m}+\frac{H\rho }{\rho^{2}-1}\;,\;\mathrm{where}\;\mathrm{the}\;\mathrm{bandwidth}\;H=\nu_{+}-\nu_{-}$$

Plots of emission maxima (λ_max_) versus peak width can be used to assess the environmental heterogeneity of the emitting tryptophan species [48]. In a homogeneous environment, the plot should fall roughly on a straight line that has been experimentally determined using free zwitterionic tryptophan in various solvents with different degrees of hydrophobicity [48,49]. A plot of our data at different lipid concentrations reveals that at the highest lipid concentration (where tryptophan fluorescence is blue-shifted due to membrane interactions), where all alpha-synuclein variants except for the A30P/V70P double mutant reaches saturation binding (Figure 3B), the emission spectrum of tryptophan falls on the line indicating a homogeneous population in a fully hydrophobic environment (Figure S2B, leftmost data points). As expected, at intermediate lipid concentrations, our data lie above the empirical line, signifying a higher-than-expected width compared to a homogeneous species, consistent with a heterogeneous population made up of free-state and bound-state species. Interestingly, the free-state spectra also indicate heterogeneity (Figure S2B, rightmost data points), likely resulting from the fact that the N-terminal ~10 residues of N-terminally acetylated alpha-synuclein sample partly helical conformations [50,51,52], resulting in a mixture of conformational states with varying degrees of solvent accessibility at position 4.

In order to plot the fluorescence-monitored binding curves as the change in bound fraction of the protein at different lipid concentrations, the bound fraction was estimated for each lipid concentration. Since the time scales of protein–membrane interactions are much slower than the fluorescence measurement time scale, spectra at intermediate lipid concentrations were fit to a linear combination of two spectra: free-state protein spectrum (no lipids) and fully bound state protein spectrum (at the highest concentration of lipids) (Figure S3). In the case of the A30P/V70P/F4W mutant, which did not reach saturation binding even at the highest lipid concentration, the WT/F4W spectrum with 10 mM lipids was used instead. Fits of the resulting curves to a bimolecular binding model between protein molecules and binding sites on lipid vesicles [53], in which the binding site concentration is related to the total lipid concentration by a proportionality constant, B_max_ (maximum binding sites per lipid molecule; 1/B_max_ is then the minimum number of lipids per biding site), were performed using an R implementation of quadratic programming to solve for the bound fraction. The minimum number of lipids per binding site, 1/B_max_, was determined for the WT protein as the lipid concentration divided by the bound protein concentration, the latter of which was determined from the NMR intensity plots (Figure 2C) as the fraction of bound protein, defined as the median value for the bound fraction of the N-terminal 9 residues, multiplied by the total protein concentration. The conditions under which the NMR intensity plots were obtained (2.5 mM lipid concentration) are in the saturating regime (Figure 3B), where every binding site on the vesicle surface is occupied by a protein molecule.

### 2.6. Exocytosis

RBL-2H3 cells were cultured as monolayers in minimal essential medium (Invitrogen Corp, Carlsbad, CA, USA) with 20% fetal bovine serum (Atlanta Biologicals, Atlanta, GA, USA) and 10 µg/mL of gentamicin sulfate (Invitrogen), as previously described [54]. Adherent cells were harvested by treatment with Trypsin-EDTA (0.05%) for 8–10 min 3–5 days after passage. RBL-2H3 cells are continuously cultured in the Baird–Holowka laboratory, routinely checked for normal function, and frozen stocks are thawed for fresh cultures as warranted.

A pcDNA 3.0 vector for cell expression of human WT alpha-synuclein was obtained as a gift from Dr. Chris Rochet (Purdue University). Plasmids for cell expression of alpha-synuclein mutants (A30P, V70P, A30PV70P, 3AE, 4G) were created from this vector by site-directed mutagenesis using Phusion High-Fidelity DNA Polymerase (New England Biolabs, Ipswich, MA, USA), and all mutations were confirmed by DNA sequencing. Plasmids for cell expression of VAMP8-pHluorin and mCherry-Rab11 were created as previously described [55,56].

RBL-2H3 cells were harvested 3–5 days after passage, and $5\times 10^{6}$ cells were suspended in 0.5 mL of cold electroporation buffer (137 mM NaCl, 2.7 mM KCl, 1 mM MgCl_2_, 1 mg/mL glucose, 20 mM HEPES, pH 7.4). Co-transfections used a reporter plasmid DNA (5 µg VAMP8-pHluorin), together with 5 µg (low expression) or 25 µg (high expression) of human alpha-synuclein (or empty vector) plasmid DNA. We found previously that cells transfected with the two constructs express both or none, such that the fluorescent VAMP8-pHluorin construct could be used as a reporter for cells co-transfected with the non-fluorescent alpha-synuclein construct [57].

For all conditions, cells were electroporated at 280 V and 950 µF using Gene Pulser X (Bio-Rad, Hercules, California, USA). Then, cells were immediately resuspended in 6 mL of medium and cultured for 24 h to recover; the medium was changed after live cells became adherent (1–3 h). For exocytosis experiments, the cell suspensions were added to three different MatTek dishes (2 mL/dish) (MatTek Corporation, Ashland, MA, USA) for recovery.

After the electroporation recovery period and prior to imaging, cells were washed once and then incubated for 5 min at 37 °C with buffered saline solution (BSS: 135 mM NaCl, 5 mM KCl, 1 mM MgCl_2_, 1.8 mM CaCl_2_, 5.6 mM glucose, 20 mM HEPES, pH 7.4). VAMP8-pHluorin fluorescence was monitored for 20 s prior to addition of 250 nM thapsigargin, and after 6–8 min of stimulation, 50 mM NH_4_Cl was added. Cells were monitored by confocal microscopy (Zeiss 710) using a heated, 40× water immersion objective. VAMP8-pHluorin was excited using the 488 nM line of a krypton/argon laser and viewed with a 502–551 nm band-pass filter. Representative movies for a control and for experiments with the WT, 4G and 3AE mutants are available upon request, and 3AE mutants are included as Supplementary Material.

Offline image analysis was conducted using Fiji ImageJ [58]. Regions of interest (ROIs) were manually drawn around individual cells from which time traces of VAMP8-pHluorin fluorescence were obtained. In case of stage movement, the ROIs were translated accordingly. For each cell, the average fluorescence of 5 frames each of before stimulation ($F_{\mathrm{basal}}$), after peak of stimulated exocytosis ($F_{\mathrm{stimulated}}$) and after NH_4_Cl administration ($F_{\mathrm{total}}$) were used to measure fraction of vesicles exocytosed (Exo) using the following equation:

(4) $$Exo=\frac{F_{\mathrm{stimulated}}-F_{\mathrm{basal}}}{F_{\mathrm{total}}-F_{\mathrm{basal}}}$$

For each condition (variant and expression level), outliers in the measured per-cell exocytosis values were excluded using the 1.5 times interquartile range method before comparison and statistical tests for significance. Analysis of the data without outlier removal did not impact the statistical comparisons between the conditions.

### 2.7. Distribution of Recycling Endosomes

RBL-2H3 cells were prepared and electroporated as described above with 5 µg of mCherry-Rab11 plasmid DNA to label recycling endosomes [56] and either 5 µg or 25 µg of alpha-synuclein (WT or mutant) or 3 µg of control plasmid DNA. Samples were fixed and immunostained with anti-alpha-synuclein antibody 3H2897 (Santa Cruz Biotechnology Cat# sc-69977) after the recovery period and imaged confocally using a 63× oil immersion objective, selecting a plane near the middle of the cell including both plasma membrane and perinuclear regions. Fluorescence from immunostained alpha-synuclein was used to select sufficiently bright cells, and mCherry-Rab11 fluorescence was quantified and used to determine the relative distributions of REs using Fiji ImageJ [58]. Fluorescence in a shell of ~800 nm thickness, drawn to include the plasma membrane and a small region extending inward, was divided by the total cellular mCherry-Rab11 fluorescence to yield the percentage of REs proximal to the plasma membrane.

### 2.8. Expression Levels of Alpha-Synuclein Using Immunocytochemistry

To directly assess protein expression levels, we quantified and compared alpha-synuclein immunostaining under conditions of high expression for the WT and linker mutants in RBL-2H3 cells (Figure S11). All three variants exhibited similar expression levels, confirming that the linker mutants are not aberrantly expressed or degraded. In addition, for the 4G mutant, we compared immunostaining at low and high expression levels, confirming an approximately 3- to 5-fold difference between the two conditions, as previously reported for the WT and other variants including A30P and V70P [57]. We previously demonstrated that VAMP8 fluorescence is a highly reliable reporter of alpha-synuclein expression levels in our system [57]. Having established by immunostaining that both the linker mutants express normally, we also visually examined VAMP8 fluorescence levels in each individual experiment to confirm they were consistent with the expected high or low expression levels.

### 2.9. Effects of Alpha-Synuclein on Enrichment of Lipid Vesicles in Synapsin-1 Condensates

The construct for expression of human synapsin-1-eGFP in mammalian cells (pEGFP-C1 vector) was a kind gift from Dragomir Milovanovic and Pietro De Camilli (Yale University). Full-length synapsin-1-eGFP was expressed in Expi293 cells and purified as described [59]. Briefly, after transfection of cells, proteins were expressed for 3 days and harvested/lysed in buffer A (25 mM Tris-HCl at pH 7.4, 300 mM NaCl, 0.5 mM TCEP) with requisite protease inhibitors. To clarify lysates, samples were spun at 17,000× *g* and filtered. Samples were then run through a nickel column (His60 Ni Superflow Resin, Takara Biosciences, San Jose, CA, USA) and eluted with Buffer A + 400 mM imidazole. Samples were then run through a size exclusion column (HiLoad 16/600 Superdex 200 pg, Cytiva Life Sciences, Marlborough, MA, USA) in Buffer B (25 mM Tris-HCl at pH 7.4, 150 mM NaCl, 0.5 mM TCEP) and subsequently concentrated. All purification steps took place at or near 4 °C.

Rhodamine-labeled SUVs were prepared as described above but using a molar ratio of DOPC/DOPE/DOPS/lissamine-rhodamine PE = 60:24:15:1 and rehydrated using the reaction buffer (25 mM Tris-HCl, 150 mM NaCl, pH 7.4). Unlabeled N-terminally acetylated WT alpha-synuclein, prepared as described above, was reconstituted from lyophilized stock in reaction buffer (25 mM Tris-HCl, 150 mM NaCl, pH 7.4). PEG-8000 was added from a stock concentration of 40% (*w*/*v*) in H_2_O. The glass slides and cover glasses were chemically cleaned, aminosilanized and passivated by reacting with NHS ester-modified PEG, as described elsewhere [60]. Microchambers were fabricated using a spacer tape between glass slides and cover glasses, through which the sample could be imaged. Phase separation of synapsin-1 in the absence or presence of 0.5 mM SUVs was initiated by adding PEG-8000 at a final concentration of 4% (*w*/*v*) to reaction buffer (25 mM Tris-HCl, 150 mM NaCl, pH 7.4). Matched samples with and without N-terminally acetylated alpha-synuclein (200 µM), prepared in independent triplicates, were imaged using Zeiss LSM-880 laser scanning confocal microscope, with excitation wavelengths of 488 nm and 532 nm for eGFP and rhodamine channels, respectively. Detector gain was adjusted to minimize saturation of the rhodamine signal to allow quantitation.

Droplet images were analyzed using Fiji-ImageJ as follows. Droplets where rhodamine signal reaches saturation were manually removed. A Gaussian blur of 2 pixels was applied to the eGFP channel, followed by background subtraction using a rolling ball diameter of 40 pixels (5.27 µM), chosen to be larger than the size of the largest droplet so as to capture background features while excluding droplets. Thresholding of the eGFP channel was performed using the Li algorithm [61]. Mean rhodamine fluorescence of all particles of area greater than 0.2 square microns (to reduce noise from off-focus or very small droplets) within the eGFP-thresholded regions was measured. Mean rhodamine fluorescence outside of droplets was determined using an inverted threshold, and a partition coefficient for SUVs in each droplet was calculated as the ratio of mean rhodamine fluorescence inside and outside each droplet. To assess statistical significance, partition coefficient values were log-transformed, and outliers were removed based on interquartile range method. Upon assessing and verifying the near-normality of the transformed data using Shapiro–Wilk test, Student’s t-test was performed to reject the null hypothesis that there was no significant difference between partition coefficient of SUVs inside synapsin-1 droplets in absence and presence of 200 µM alpha-synuclein. The difference between the two conditions remained highly significant (*p* < 0.001) when the data were analyzed without outlier removal.

## 3. Results

### 3.1. Linker Mutants Bias Linker Region Helicity in Micelle-Bound State

**Figure 1:**
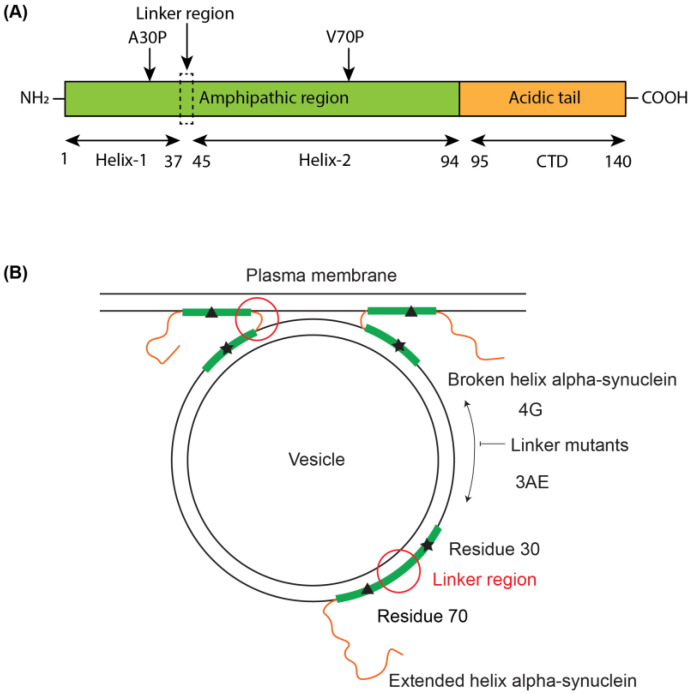
Schematic illustrating the design logic for alpha-synuclein linker mutants. (**A**) Domain structure of alpha-synuclein and location of mutations used in the study. (**B**) Proposed functional contexts of different membrane-bound conformations of alpha-synuclein and the effects of the two linker region mutations, 4G and 3AE, on these conformations. The helix-1 and helix-2 regions are depicted as green rectangles and the linker region in the broken-helix state and the disordered C-terminal tail as orange lines. The linker region is highlighted with a red circle in both conformations, and the positions of the A30P and V70P mutations are marked with a star and a triangle, respectively. The two linker regions mutations are expected to inhibit conformational exchange and bias the membrane-bound conformation of the protein towards the broken-helix (4G) or extended-helix (3AE) state.

**Figure 2:**
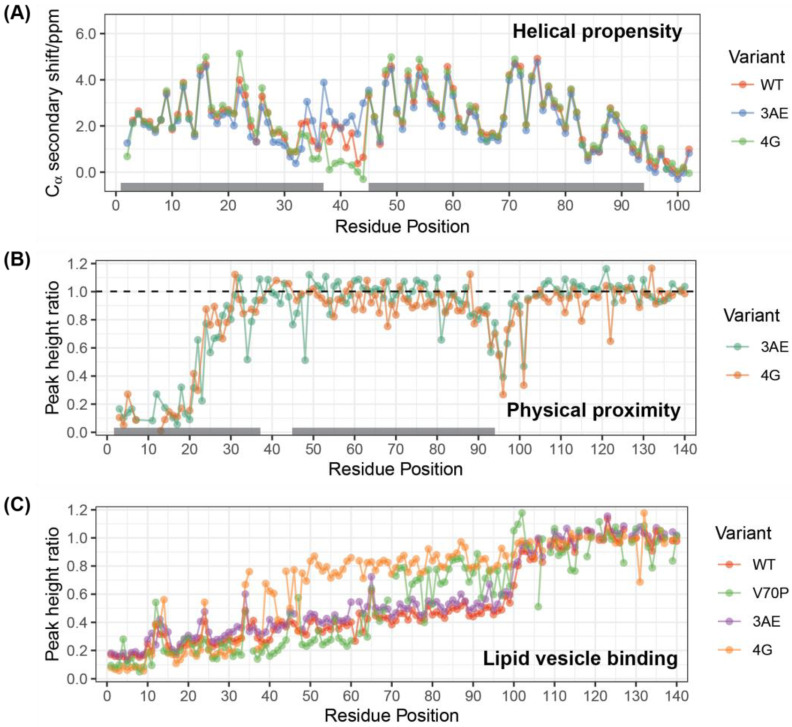
Effects of the linker region mutations on the micelle- and vesicle-bound states of alpha-synuclein. (**A**) NMR C-alpha secondary chemical shifts for micelle-bound WT alpha-synuclein and the 4G and 3AE mutants. Positive secondary shifts above ~1 PPM are indicative of significant helical propensity. Deuterated SDS concentration was 40 mM, and protein concentrations were 100–200 µM. Data were collected at 40 °C. (**B**) PRE in micelle-bound alpha-synuclein 4G and 3AE mutants labeled with a paramagnetic spin-label at position 9. SDS concentration was 40 mM, and protein concentrations were 100 µM. Data were collected at 40 °C. (**C**) Intensity ratios of signals from NMR ^15^N-^1^H HSQC spectra of 50 µM alpha-synuclein variants obtained in the presence vs. the absence of SUVs at a total lipid concentration of 2.5 mM. Data were collected at 10 °C.

We designed two conformationally selective mutants of alpha-synuclein in which we mutated residues 39 to 42 in the linker region from YVGS to either AAAE (3AE mutant) or GGGG (4G mutant) [62]. The mutants were designed to preferentially populate the extended-helix (3AE) or the broken-helix (4G) conformations (Figure 1). Alanine and glutamine have higher helical propensity than tyrosine, valine and serine, while glycine exhibits very low helical propensity [63,64]. We then investigated the structure of the 3AE and 4G mutants when bound to membrane-mimetic detergent micelles using solution-state NMR spectroscopy. C_α_ secondary shifts for the SDS micelle-bound linker region mutants show that the 3AE mutant features a higher helical propensity in the linker region, while the 4G mutant exhibits a more extensive break between helix-1 and helix-2 than the WT protein, with no effect observed outside the linker region (Figure 2A). Paramagnetic relaxation enhancement (PRE) measurements using MTSL labeling via an S9C mutation [25] were similar for the 3AE, 4G and WT proteins (Figure 2B), indicating proximity (within ~25 Å [65,66]) of the N- and C-termini of helix-1 and helix-2, respectively, for all three variants and suggesting that this proximity does not require a break in the helical structure, consistent with a previous report [67].

### 3.2. 4G Mutant Prevents Propagation of the Vesicle-Bound Extended-Helix Conformation

Binding of alpha-synuclein to small unilamellar vesicles (SUVs) results in a fractional decrease of NMR signal intensities corresponding to the bound fraction for each residue involved in the interaction. Peak intensity ratios in the presence vs. absence of SUVs therefore report on the free state fraction for each position in the protein. Intensity ratio plots for 50 µM WT N-terminally acetylated alpha-synuclein in the presence of 2.5 mM lipid SUVs (Figure 2C, red) reveal that the bound fraction is maximal at the very N-terminus of the protein and gradually decreases towards the C-terminus of the lipid-binding domain, reflecting a consensus in the field that lipid binding proceeds from the N- to the C-terminus of the lipid-binding domain [68,69]. The 3AE mutant shows similar lipid-binding behavior to the WT protein (Figure 2C, purple). In contrast, the 4G mutant shows a distinct break in its lipid-binding profile, with residues 34–94 exhibiting higher intensity ratios compared to residues 1–33 (Figure 2C, orange). We previously observed a similar break in the vesicle-binding profiles of alpha-synuclein mutant V70P, which introduces a proline in the middle of helix-2. Interestingly, the intensity ratios at the very N-terminus are lower for the 4G and V70P (Figure 2C, green) mutants than for the WT or 3AE variants, suggesting that this apparently enhanced binding may be a common effect of truncating the extended-helix conformation. This effect could potentially result from a smaller binding footprint on the membrane surface, resulting in a greater number of available binding sites.

### 3.3. Linker Mutants Alter Effects of Alpha-Synuclein on Vesicle Release

We performed stimulated exocytosis assays in RBL-2H3 cells as described earlier [57]. We used RBL-2H3 rat basophilic leukemia cells as a model system to quantify Ca^2+^-stimulated exocytosis of recycling endosomes labeled with the pH-sensitive fluorescent marker VAMP8-pHluorin. Exocytosis in these cells is stimulated by Ca^2+^ release from the endoplasmic reticulum (ER), which can be triggered by signaling downstream of antigen receptors or by inhibition of the sarco/endoplasmic Ca^2+^ ATPase (SERCA) using the SERCA inhibitor thapsigargin [70]. Cells were co-transfected with VAMP8-pHluorin and low (5 µg plasmid) or high (25 µg plasmid) levels of alpha-synuclein variants, stimulated with 250 nM thapsigargin and imaged for 400 s using a confocal microscope. Ammonium chloride was added at the end of the experiment to a final concentration of 50 mM to neutralize all vesicles in the cell interior and provide a measure of the total recycling vesicle pool. As we reported previously, low levels of WT alpha-synuclein lead to inhibition of vesicle release (Figure S1A), but high expression levels were associated with enhanced vesicle release (Figure S1B) compared to empty vector [57]. The 3AE mutant was less effective at inhibiting release at low levels and more effective at enhancing release at high levels compared to empty vector, consistent with its design goals of stabilizing the extended-helix state over the broken-helix state. However, despite favoring the broken-helix state (Figure 2A), the 4G mutant caused no inhibition of vesicle release at low expression levels, suggesting that the two flexibly coupled helices alone are insufficient for the inhibitory function of alpha-synuclein. Release at high expression levels of the 4G mutant was comparable to that for the WT protein, suggesting that the altered binding mode of this mutant was still somewhat effective at enhancing exocytosis.

**Figure 3:**
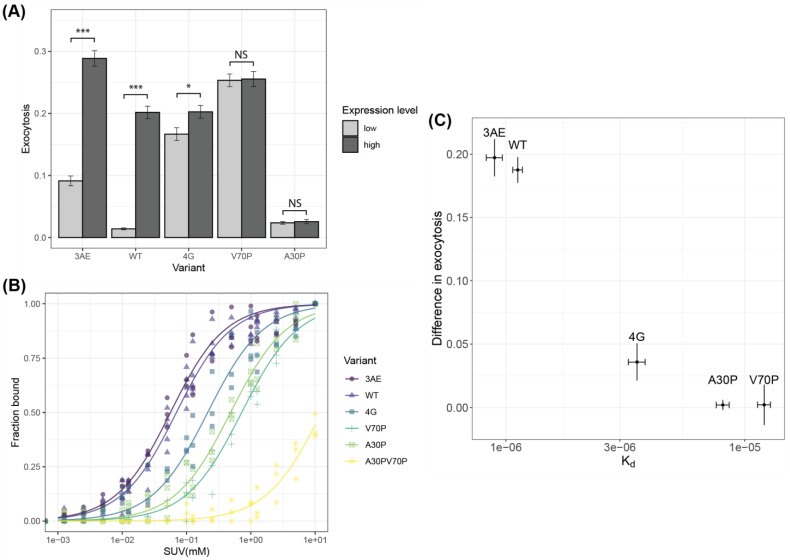
Functional assays and membrane affinity of alpha-synuclein variants. (**A**) Thapsigargin-stimulated exocytosis in RBL-2H3 cells transfected with low or high levels of WT or mutant alpha-synuclein measured using fluorescence of a VAMP8-pHlourin reporter. The difference in exocytosis levels between low and high expression levels represents the degree by which exocytosis is enhanced at high expression levels (*t*-test between high/low, *p* values *** < 0.001 < * < 0.05 < NS). (**B**) Lipid-binding curves for WT and mutant alpha-synuclein in an F4W mutant background measured by intrinsic tryptophan fluorescence as a function of increasing lipid concentrations. Protein concentrations were 0.1 µM, and lipid concentrations ranged from 1.25 µM to 10 mM. Data were acquired at a temperature of 22 °C (room temperature) by excitation at 280 nm and detection at 300–500 nm at 10 nm resolution, 60 flashes averaged and then baseline subtracted using a no-protein control, analyzed to extract bound fraction at every lipid/protein ratio and fit as described in the methods. Resulting fits are shown in solid lines. For each day of experiments with a new lipid vesicle preparation, WT data served as an internal control to account for variations. (**C**) Plot of membrane affinity derived from fits to the data in panel B vs. extent of enhancement of exocytosis derived from the data in panel A for WT and mutant alpha-synuclein.

We previously showed, using TIRF imaging of individual vesicle fusion events, that despite potentiation of vesicle release at high expression levels of alpha-synuclein, the rate of vesicle release is still lower than for the empty vector control, indicating that the inhibitory and potentiating activities of the protein are not mutually exclusive and instead operate in tandem [70]. In this case, the potentiating activity is better measured by comparing release at high levels to release at low levels, with the difference representing the isolated potentiating activity. Using this measure, we observe (Figure 3A) that the WT and 3AE variants have the greatest potentiation activity, followed by the 4G mutant. The V70P mutant exhibits release that is comparable to or slightly higher than that of the WT protein at high expression levels (Figure S1B), and we previously took this as evidence that the V70P mutant does not perturb enhancement of release [70]. However, when compared directly to its effects at low expression levels, it is evident that the V70P does not significantly enhance vesicle release. This analysis also confirmed the surprising result that the 4G mutant was able to sustain potentiation of exocytosis despite its abbreviated binding mode to lipid vesicles, while revealing that the V70P mutant, which exhibits a more extensive binding mode than the 4G mutant, does not potentiate vesicle release. These results suggest that binding interactions reflected in intensity ratio measurements may not capture all aspects of vesicle binding and led us to examine binding using additional assays. As an additional reference point, we examined the difference in release for low and high levels of the PD mutant, A30P. This mutant effectively inhibits release at low levels but showed no increase in release at higher levels (Figure 3A).

### 3.4. Membrane Affinity for Isolated Vesicles Correlates with Potentiation of Exocytosis

We previously argued that the potentiating activity of alpha-synuclein is derived from its ability to bind isolated vesicles. While lipid-binding profiles derived from NMR reveal changes in localized binding to lipid vesicles, they do not easily provide information on binding affinity since they are performed at relatively high protein and lipid concentrations. To investigate the correlation between membrane binding affinity and potentiating activity, we measured the affinity of our variants to lipid vesicles using fluorescence, following a previously reported protocol employing the introduction of a single tryptophan probe at position 4 (F4W mutation) [53] and relying on the environment-sensitive nature of tryptophan fluorescence emission spectra (Figure S2) [49,71] Notably, the F4W mutation was conclusively shown not to cause significant changes in the membrane affinity or secondary structure of vesicle-bound alpha-synuclein [53]. The sensitivity of this method allowed us to acquire data at very low (0.1 µM) protein concentrations, which enabled us to reach lipid/protein molar ratios of 100,000:1, at which point vesicle binding reached saturation for all of the mutants except one (Figure 3B and Figure S2B). Spectra at each lipid concentration were fit to a linear combination of the free-state and bound-state spectra yielding the fraction of protein in the bound state (Figure S3). Fits of the resulting binding curves to a quadratic bimolecular binding model between protein molecules and binding sites on lipid vesicles [53], in which the binding site concentration is related to the total lipid concentration by a proportionality constant, B_max_ (maximum binding sites per lipid molecule; 1/B_max_ is then the minimum number of lipids per binding site) provided estimates of the dissociation constant, K_d_. Notably, although B_max_ can in principle be fit simultaneously with K_d_, we could instead estimate B_max_ from the fraction of bound protein determined by NMR studies in the saturation regime (see Section 2), where protein is in excess of binding sites (Figure 2C). In this regime, protein molecules will bind to all available sites (protein and lipid concentrations are well above K_d_), so the presence of excess free protein indicates all sites are occupied. The amount of bound protein divided by the total lipid concentration thus provides an estimate of the number of lipids per binding sites. For N-terminally acetylated WT alpha-synuclein, we obtained a value of 59 lipid molecules per binding site, which is consistent with estimates from electron spin resonance (ESR) data using dimyristoyl phosphatidylglycerol (DMPG) membranes (36–100 lipids per binding site) [72] but significantly lower than that estimated for non-acetylated alpha-synuclein using circular dichroism spectroscopy (500 lipids per binding site) [53].

The K_d_ values we obtained from fits of the fluorescence data (Table 1) indicate that WT alpha-synuclein and the 3AE mutant bind with the highest affinity, followed by 4G and then A30P and V70P. We also examined binding by the A30P/V70P double mutant, which showed weaker binding than either A30P or V70P alone and did not reach saturation at the highest lipid concentrations (Figure 3B and Figure S2B). The K_d_ values show a correlation with the ability of the different variants to enhance vesicle release at high expression levels (Figure 3C), a result consistent with our proposal that binding to isolated vesicles is linked to the ability of alpha-synuclein to potentiate exocytosis. We previously reported that high expression of WT alpha-synuclein disperses mCherry-Rab11-labeled recycling endosomes from the endocytic recycling compartment (ERC) to the cell periphery [57]. We hypothesized that this redistribution leads to an increased abundance of vesicles readily available for fusion upon stimulation, contributing to the observed potentiation of exocytosis. Notably, the A30P mutant, which does not potentiate release at high expression levels, does not lead to a redistribution of mCherry-Rab11. Here, we examined the effects of the linker mutants on the distribution of fluorescently tagged Rab11. Both the 3AE and 4G variants showed a significant difference in exocytosis between high and low expression levels (Figure 3A), consistent with the ability of both mutants to enhance vesicle release. However, the extent of redistribution was similar for both the mutants and the WT protein (Figure S4), despite the decreased potentiating activity and vesicle affinity of the 4G mutant, indicating that RE redistribution is not linearly correlated with enhanced exocytosis or vesicle binding.

### 3.5. Alpha-Synuclein Can Disperse Vesicles from Condensates In Vitro

Our investigations of the effects of alpha-synuclein on intracellular vesicle distributions were motivated by studies demonstrating that increasing levels of alpha-synuclein correlate with increasing dispersion of SVs from SV clusters in neurons [16,24]. Recent evidence suggests that SV clusters are formed by the sequestration of SVs in synapsin-1 phase-separated condensates [59]. We explored whether alpha-synuclein could either dissolve synapsin-1-eGFP condensates in vitro or disperse lipid vesicles from synapsin condensates as a potential mechanism by which it could disperse SV clusters at presynaptic nerve terminals. As previously reported, rhodamine-labeled SUVs were preferentially partitioned in the synapsin-1-eGFP phase-separated droplets formed in the presence of polyethylene glycol (PEG) (Figure S5A). The presence of 200 µM unlabeled alpha-synuclein did not eliminate synapsin droplets (Figure S5A) but did significantly decrease the partitioning of SUVs into the droplets (Figure S5A,B). This result is consistent with a recent report describing the interactions of alpha-synuclein with synapsin condensates [73] and could provide an explanation for the dispersal of SV clusters with increasing synuclein levels. In RBL-2H3 cells, we also observe that increasing levels of alpha-synuclein disperse recycling vesicles from intracellular stores, as described above, suggesting that a similar mechanism could be involved [57].

### 3.6. Bipartite Binding Contributes to Affinity of Alpha-Synuclein to Membranes

**Figure 4:**
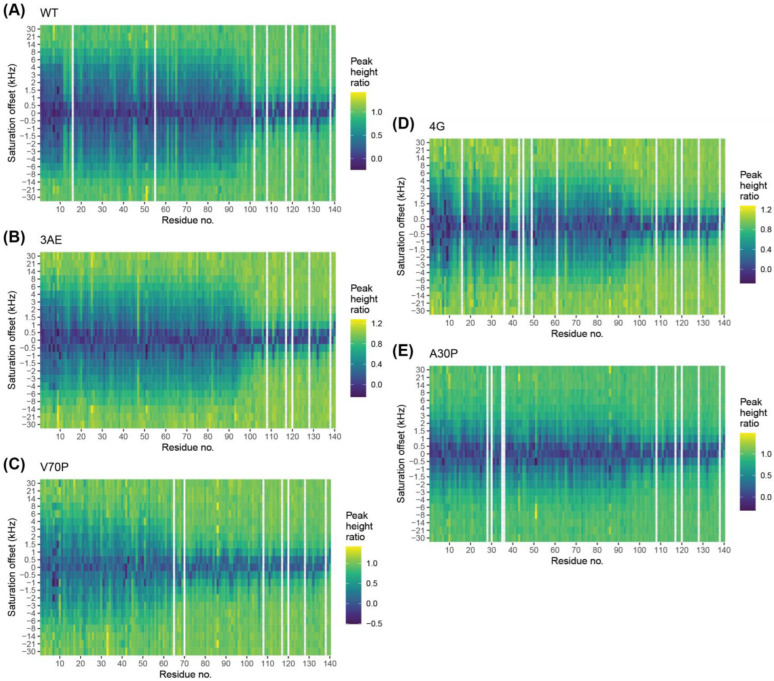
Alpha-synuclein membrane binding profiles. DEST intensity ratios as a function of saturation offset and residue number for 100 µM WT (**A**), 3AE (**B**), V70P (**C**), 4G (**D**) and A30P (**E**) alpha-synuclein with 1 mM 60:25:15 DOPC/DOPE/DOPS lipid SUVs at 13 °C, using a 700 MHz spectrometer and a saturation bandwidth of 400 Hz. Broad profiles indicate exchange with a slowly tumbling membrane-bound state while narrow profiles indicate less or no membrane interaction.

The higher affinity of the 4G mutant for membranes compared with the V70P mutant is surprising, given that our NMR intensity ratio assay indicates that a larger portion of the protein interacts with membranes for the V70P variant (Figure 2C). To examine membrane binding more closely, we performed ^15^N-DEST NMR (Dark-state Exchange Saturation Transfer) experiments that probe the exchange between the invisible vesicle-bound state and the visible free state. These experiments are performed under conditions where only a small fraction of protein is bound at any one time, and unlike the intensity ratio assay, which reports on the populations of free protein, DEST probes transient interactions of individual residues with membranes, which result in a broadening of the corresponding DEST profiles [42]. As expected, DEST profiles of 100 μM N-terminally acetylated WT alpha-synuclein in the presence of 1 mM SUVs show broadening over the entire lipid-binding domain of the protein, while residues in the C-terminal tail that do not bind strongly to membranes show narrow DEST profiles and provide an internal control (Figure 4A). The 3AE mutant had a similar DEST profile as the WT, signifying that membrane binding modes of the protein are unperturbed for this mutant (Figure 4B). The V70P mutant, which showed disruption of the lipid interaction C-terminal to position 65 in the intensity ratio assay (Figure 2C), features broadening similar to the WT protein for residues 1–65 but features narrow DEST profiles C-terminal to position 65 that are similar to those for residues in the C-terminal tail (Figure 4C), indicating that there is no significant interaction with lipid membranes beyond this position. Interestingly, the 4G mutant exhibits DEST profiles broadened to a similar extent as for the WT protein both for residues 1–35 and for residues 50–94 (Figure 4D), which correspond roughly to the helix-1 and helix-2 regions of the broken-helix state. The presence of four glycine residues in the linker region of this mutant disallows helix propagation, and such a DEST profile thus strongly suggests independent binding of the helix-2 region to the membrane surface. This binding mode is apparently too transient to result in intensity decreases under the conditions used for the intensity ratio assay in Figure 2, but it does appear to contribute to membrane affinity, as reflected in the higher K_d_ value of the 4G compared with the V70P mutant. Although the conformation of such a transiently bound state cannot be probed directly using these experiments, at higher lipid concentrations, binding of the helix-2 region of the 4G mutant can be observed using the intensity ratio assay (Figure S6). This suggests the possibility that when they are structurally decoupled, the helix-1 region of alpha-synuclein outcompetes the helix-2 region for membrane binding sites. This is consistent with a recent computational study that reports binding of the helix-2 region to membranes in both helical and disordered conformations with a lower affinity than the helix-1 region [74].

We also examined the DEST profiles of the PD mutant A30P in the presence of SUVs (Figure 4E). As previously reported, the DEST profiles are not as broad as those for the WT protein, consistent with a decrease in local binding to membranes throughout the protein sequence for this mutant. Furthermore, as for the 4G mutant but different from the V70P mutant, binding is evident beyond the site of mutation throughout the remainder of the lipid-binding domain. This observation was also interpreted previously as evidence for independent binding by the helix-2 region, but it is not clear to what extent the single proline mutation at position 30 decouples helix formation between the helix-1 and helix-2 regions, since the local helical structure of the micelle-bound A30P mutant is only perturbed for a few turns and resumes before the linker region [75]. Furthermore, the total helical content of this mutant when bound to vesicles at high lipid concentrations is only slightly decreased compared to the WT protein [76].

### 3.7. Quantitative Analysis of DEST Data Support Bipartite Binding Model

Quantitative fits to DEST data can be used to extract information regarding the kinetics of membrane binding and release and the ensemble of membrane-bound conformations at the level of individual residues [42,77]. To apply the model developed by Fawzi et al., we first examined whether binding of alpha-synuclein to vesicles is consistent with a pseudo-first-order process. We showed that increases in R_2_, which can be used as an estimate of the rate of membrane association, depend on lipid concentration but are relatively independent of protein concentration (Figure S7), confirming that binding can be modeled as a pseudo-first-order process. Next, we applied the DESTfit analysis package developed by Fawzi et al. to fit our DEST and ^15^N R_2_ relaxation data to different binding models [42,77]. Since the C-terminal tail does not bind to lipids, we only considered the lipid-binding domain (residue 1–98) for the fitting. The models, as described by Fawzi et al. [77], include a simple two-state model for membrane binding, where the protein exchanges between a single free state and a single bound state for each residue, and a pseudo-two-state model, where for each residue, bound-state conformations are divided into two subsets, those in which the residue is in direct contact with the vesicle surface or those where it is tethered to the vesicle surface by other nearby residues that are in direct contact (see Section 2). Notably, modeling multiple bound-state conformations allows for different bound-state relaxation properties, better accounting for variations in the shape of the DEST profiles.

The success of each model in fitting the data was assessed by its ability to reproduce the difference in R_2_ measured in the absence and presence of vesicles. For the WT protein, the two-state model did not provide a good fit, while the pseudo-two-state model resulted in adequate fits over the entire sequence (Figure S8). This was also observed to be the case for the 3AE, the 4G and the A30P mutants. For each of these variants, the fit results suggest a significant population of direct contact binding modes for residues in the helix-2 region of the protein (Figure S9), consistent with an independent binding mode for this region. Interestingly, at the N-terminal region of each of these variants, the direct contact population becomes smaller, suggesting that tethered binding modes dominate in this region. The N-terminal region of alpha-synuclein is known to be the tightest binding region of the protein, as seen in our intensity ratio plots and as documented in other studies. Because of tight binding in this region, the local off-rate is expected to be quite slow, likely smaller than the longitudinal relaxation rate R_1_, enabling only a fraction of direct contact binding events to contribute to the DEST profiles in this region. In addition, the tethered state R2 rates extracted from the model are much higher in this N-terminal region compared with the helix-2 region, consistent with tighter binding of this site even in tethered binding modes. Unlike for the other variants, the DEST data for the V70P mutant could be fit using the simpler two-state model, which does not require heterogeneous binding modes. This is consistent with the lack of independent binding of the helix-2 region of this mutant. The bound-state R2 values extracted from this model are consistent with the directly bound R2 values estimated for the other variants and an order of magnitude larger than those estimated for the tethered binding modes of the other variants, confirming that this binding mode corresponds to a direct contact mode.

## 4. Discussion

While the normal function(s) of alpha-synuclein remain incompletely understood, considerable evidence indicates that the protein can function in the regulation of the SV cycle by influencing endocytosis [78,79,80,81], intracellular vesicle pools [8,16,24,82] and exocytosis [16,17,83,84,85,86]. The structural underpinnings of these functions are poorly understood, despite considerable progress in delineating the different conformations that alpha-synuclein can adopt in vitro. We recently developed a structure–function assay for the effects of alpha-synuclein on vesicle exocytosis utilizing a model cell line, RBL-2H3, which provides facile stimulation of vesicle release that can be monitored conveniently via confocal fluorescence microscopy using the pHluorin assay [57]. Our studies revealed that low levels of alpha-synuclein expression are sufficient to inhibit the release of recycling endosomes triggered by thapsigargin or antigen treatment, while TIRF microscopy measurements of individual vesicle fusion events indicated that this inhibition was likely operating directly at the level of individual fusion events by reducing the probability of fusion. We identified a mutation, V70P, that abrogated the ability of alpha-synuclein to inhibit release in this assay and proposed that this mutation prevented the protein from bridging between the vesicle and plasma membranes via a broken-helix configuration that we posit is required for inhibitory activity [57]. In this conformation, we [25,87,88,89,90] and others [91] have proposed that the two helices of alpha-synuclein are thought to preferentially bind to either the vesicle membrane or inner plasma membrane, creating a membrane-bridging conformation.

Surprisingly, we observed that higher levels of alpha-synuclein expression lead to increased levels of vesicle release compared with empty vector controls [57]. Increased release was associated with a redistribution of mCherry-Rab11 staining, a marker for the endocytic recycling compartment from which recycling endosomes originate, from the interior of the cell to the vicinity of the plasma membrane. This suggested that increased release was associated with an increased pool of vesicles near the membrane. Supporting this hypothesis, the A30P mutant of alpha-synuclein, which was known to be defective in binding to isolated membrane vesicles, abrogated increased vesicle release at high expression levels and also eliminated the redistribution of Rab11 to the cell periphery. Since alpha-synuclein binds to isolated vesicles predominantly in an extended-helix conformation, we proposed that this binding mode is required for potentiation of vesicle release.

Although alpha-synuclein is a neuronal protein, our previous studies demonstrated that RBL-2H3 cells are advantageous for structure–function analyses of this protein because, as described above, they allow us to decouple the effects of alpha-synuclein on the fusion of docked vesicles from its effects in intracellular vesicle pools and distribution. These effects are difficult to deconvolute in neurons, where vesicles can only fuse at the active zone, whereas in our model cells vesicles can fuse anywhere on the plasma membrane. Here, we designed two new mutants of alpha-synuclein, designed to bias the membrane-bound conformation of the protein towards either the broken-helix state (4G mutant), which we posited should favor the inhibitory activity of the protein, or the extended-helix state (3AE mutant), which we posited should favor the potentiating activity of the protein. The 3AE mutant of alpha-synuclein indeed favors the extended-helix over the broken-helix state. As expected, this mutant does not inhibit vesicle release as effectively as the WT but is fully able to potentiate release. These results support the model in which the broken-helix state mediates direct inhibition of release, while the extended-helix state characteristic of binding to isolated vesicles mediates enhanced release. Surprisingly, despite favoring the broken-helix conformation, the 4G mutant was also able to potentiate vesicle release, although to a lesser extent than the WT and 3AE variants. We reasoned that the circumscribed binding mode of the 4G mutant to isolated vesicles was sufficient to confer a weak potentiating activity. However, the V70P mutant, which exhibits a vesicle binding mode that is more extensive than that of the 4G mutant but less than that of the WT protein, did not exhibit detectable enhancement of vesicle release. To investigate this apparent discrepancy, we measured the binding affinity of the different variants to vesicles using intrinsic tryptophan fluorescence spectroscopy. The results revealed that the 4G mutant in fact binds to vesicles more tightly that the V70P mutant, suggesting an explanation for its ability to enhance vesicle release. Indeed, a plot of the lipid binding affinity of the four different alpha-synuclein variants versus their potentiating activity indicates a correlation between these two properties.

We then investigated the basis for the higher affinity of the 4G mutant for vesicles, compared with the V70P mutant. Using DEST, we showed that the 4G mutant binds to vesicles using both the helix-1 and helix-2 regions. Binding in the helix-1 region occurs on a longer time scale and can be observed in equilibrium experiments, while binding by the helix-2 region is more transient. In contrast, the V70P mutant only features a single binding mode and is not capable of independent binding via its helix-2 region, presumably because the proline residue situated in the middle of helix-2 precludes this binding mode. Previous reports had also suggested that the helix-2 region of alpha-synuclein can bind to membranes independently based on DEST studies of the A30P mutant [92]. However, a single proline is not fully effective at interrupting helical structure, so the A30P mutation may not completely decouple helix propagation and membrane binding in the helix-2 region from helix-1. In contrast, propagation of helical structure across a four-glycine linker would be extremely unfavorable, so our observation of independent binding of the helix-2 region in the 4G mutant provides clear evidence of independent membrane binding in this region. A recent publication also concluded, based on a combination of biophysical measurements, that independent binding of the N-terminal region and the helix-2 regions of alpha-synuclein both contribute to its membrane binding affinity [69].

Surprisingly, despite creating a clear break between helix-1 and helix-2, the 4G mutant resulted in a loss of direct inhibition of vesicle release by alpha-synuclein. This result indicates that the ability to form two separate helices is not, by itself, sufficient for the inhibitory activity of alpha-synuclein. Further studies will be required to delineate what additional features are required for this activity. Since the 4G mutant effectively decouples propagation of helical structure across the linker region, it may be that some degree of structural coupling between the helix-1 and helix-2 regions is required for alpha-synuclein to directly inhibit vesicle release.

Inhibition of vesicle secretion by alpha-synuclein overexpression has been previously reported in neuronal cells and has been attributed in part to a reduction of intracellular vesicle pools [16]. Indeed, increasing levels of alpha-synuclein lead to a disruption of SV clustering [16,24]. In neurons, unlike in less specialized cells, vesicles must fuse with the plasma membrane at specialized sites known as active zones, which are closely apposed to SV clusters. Dispersion of vesicles from their clusters in such cells would be expected to lead to a reduction in vesicle release as vesicles are removed from the proximity of the active zones. Thus, while high levels of alpha-synuclein appear to cause a redistribution of vesicles out of internal pools in both neuronal cells and in RBL-2H3 cells, in the latter this leads to an increase in release as more vesicles are available at the outer cell membrane for fusion, while in neurons this leads to a decrease in release, as vesicles are mislocated away from the requisite sites of fusion.

The mechanisms by which alpha-synuclein may disperse vesicles from internal stores remains unclear, but the clustering of SVs was recently proposed to be mediated by the sequestration of vesicles in condensates formed by the presynaptic protein synapsin-1. We proposed previously that alpha-synuclein could interfere with this process either by interfering with synapsin-1 condensate formation or by release vesicles from such condensates [57]. Here, we investigated the effects of alpha-synuclein on synapsin-1 condensates with and without vesicles in vitro. Under our conditions, alpha-synuclein does not dissolve synapsin-1 condensates but does significantly reduce the degree to which fluorescently labeled vesicles co-localize with synapsin-1 droplets. Hence, alpha-synuclein appears to disperse vesicles from condensates, resulting in their release and mislocalization at presynaptic nerve terminals. A recent report also examined the interplay between alpha-synuclein and synapsin condensate formation [73], reporting that synuclein is recruited into synapsin condensates. This observation is complementary to, but consistent with, our own results, in that synuclein entry into synapsin condensates is likely required for its ability to release vesicles. This same study also reported that vesicles accelerate the formation of synapsin-vesicle condensates, while excess alpha-synuclein retards this process. Condensate formation was assessed by turbidity, and the vesicle content was not directly measured, but our results suggest that this effect of alpha-synuclein likely originates from a decrease in the number of vesicles within the forming condensates, leading to the reduced rate of condensate formation. Our observation that alpha-synuclein disperses vesicles from the endocytic recycling compartment led us to suggest that the mechanism involved may be similar and that the ERC may also be comprised, at least in parts, of protein-membrane condensates. Future work will be required to address this intriguing hypothesis.

## Figures and Tables

**Table 1:** K_d_ values for the different mutants of alpha-synuclein extracted from fits to a biomolecular reaction between protein molecules and binding sites on the vesicle surface, where the total concentration of binding sites was taken to be B_max_ times the lipid concentration, and B_max_ was determined from NMR intensity ratios as described in the Section 2.

Variant	K_d_	SEM	*p*-Value
**WT**	$1.12\times 10^{-6}$	$5.22\times 10^{-8}$	$7.98\times 10^{-36}$
**A30P**	$8.09\times 10^{-6}$	$5.01\times 10^{-7}$	$1.74\times 10^{-21}$
**V70P**	$1.21\times 10^{-5}$	$7.52\times 10^{-7}$	$2.36\times 10^{-21}$
**A30P/V70P**	$2.13\times 10^{-4}$	$1.41\times 10^{-5}$	$3.25\times 10^{-20}$
**3AE**	$8.96\times 10^{-7}$	$6.92\times 10^{-8}$	$1.39\times 10^{-17}$
**4G**	$3.54\times 10^{-6}$	$2.87\times 10^{-7}$	$9.22\times 10^{-17}$

## Data Availability

NMR backbone chemical shift assignments for SDS micelle-bound alpha-synuclein 3AE and 4G variants have been deposited in the BMRB database (BMRB accession numbers 51632 and 51633, respectively). All other data are available upon request to David Eliezer (dae2005@med.conell.edu).

## Supplementary Materials

The following supporting information can be downloaded at: https://www.mdpi.com/article/10.3390/biom12121816/s1. Eleven supplementary figures with the following titles and legends. Figure S1: Effects of alpha-synuclein variants on stimulated exocytosis at (A) low and (B) high expression levels. Thapsigargin-stimulated exocytosis of RBL-2H3 cells, quantified using VAMP8-pHluorin fluorescence time-course imaging of individual cells, for alpha-synuclein variants transfected at low (left panel) and high (right panel) concentrations. After removal of outliers (see Section 2), statistical significance was determined using one-way analysis of variance followed by pairwise p-values determined by Tukey’s post hoc test. Individual data points used for statistical analysis are shown as blue circles; outlier values within the plotting range are shown as red circles. Significance levels: p values *** < 0.001 < ** < 0.01 < * < 0.05 < NS. Error bars represent SEM; Figure S2: Membrane binding monitored using intrinsic tryptophan fluorescence. (A) Alpha-synuclein extended-helix structure schematic showing location of a single tryptophan residue that was incorporated at position 4 (F4W mutation) of N-terminally acetylated alpha-synuclein variants to serve as an environment-sensitive fluorescence reporter. (B) Fluorescence spectra were collected at 10 nm resolution from proteins at 0.1 µM concentration with variable concentrations of lipid SUVs. After subtracting concentration-matched lipid-only spectra to account for scattering background, the background-subtracted spectra (filled points) were fit to a log-normal model [45,46,47] (solid lines). (C) Plots of peak position versus full width at half maxima for each fitted spectrum. The solid line represents the empirical linear correlation between peak position and peak width for zwitterionic tryptophan in solvents with decreasing degrees of hydrophobicity, plotted as *y* = −156.7 + 0.624 *x* [48]. Deviations from the line indicate heterogeneity in the hydrophobicity of the environment experienced by Trp probe, which may reflect a mixture of bound and unbound states; Figure S3: Fitting of individual tryptophan fluorescence spectra as a linear combination of free-state and fully bound state spectra. Each background-subtracted spectrum at intermediate lipid concentrations (e.g., at 0.025 mM lipid, cyan spectrum, *S_i_*) was fit as a linear combination of the free-state spectrum (red, *S_free_*) and the fully bound-state spectrum at the highest lipid concentration (10 mM, green, *S_bound_*), using the equation *Fitted S_i_* = *c*_1_. *S_free_* + *c*_2_. *S_bound_*. The bound fraction at every lipid concentration was determined using the fitted coefficients as $\frac{c_{2}}{c_{1}+c_{2}}$; Figure S4: Redistribution of recycling endosomes at high expression levels of alpha-synuclein variants. mCherry fluorescence from mCherry-Rab11 was used as a measure of recycling endosomes. For individual cells, fluorescence integrated over a membrane-proximal zone, defined as a shell of ~800 nm thickness from the cell membrane, was divided by the total mCherry fluorescence to provide a measure of the fraction of membrane-proximal recycling endosomes. An increase in the membrane-proximal fraction in the presence of high levels of alpha-synuclein compared with control indicates a redistribution of recycling endosomes from the cell interior to the plasma membrane. The box represents 1st to 3rd quartile of the data, the midline represents the median value, and the cross represents the mean value. Significance levels: *p* values *** < 0.001 < ** < 0.01 < * < 0.05 < NS; Figure S5: Effect of wild-type alpha-synuclein on enrichment of small unilamellar vesicles in synapsin-1 phase-separated droplets: (A) Colocalization of rhodamine fluorescence from rhodamine-labeled SUVs (0.5 mM) with eGFP fluorescence from synapsin-1-eGFP phase-separated droplets in the absence (upper panels) and presence (lower panels) of N-terminally acetylated wild-type alpha-synuclein (200 µM) was used to assess the enrichment of vesicles in synapsin droplets. Representative micrographs are shown. All images were acquired using the same image acquisition and processing parameters. Scale bar: 10 µM. (B) Quantification of vesicle enrichment inside each droplet. Blue circles represent data points used for statistical analysis; red circles represent outlier values. In the box plot, the central line represents the median, the boundaries of the box represent the 25th and 75th percentile values and the ends of the vertical line represent the range of the data. Statistical significance was determined by t-test of normally distributed log-transformed data. *** *p* < 0.001; Figure S6: SUV binding of N-terminally acetylated alpha-synuclein 4G mutant with increasing concentrations of lipid SUVs. The peak height ratio, representing the free fraction per residue, was determined by ratio of ^1^H-^15^N HSQC resonances between samples with and without SUV. Data were collected at 50 µM protein concentration and 10 °C; Figure S7: Dependence of N-terminally acetylated WT alpha-synuclein ΔR_2_ on lipid and protein concentration. (A) The difference in R_2_ relaxation rates in presence versus absence of SUVs (ΔR_2_) for 100 µM N-terminally acetylated WT alpha-synuclein with different concentrations of lipid SUVs. (B) The difference in R_2_ relaxation rates in presence versus absence of SUVs (ΔR_2_) for different concentrations of N-terminally acetylated WT alpha-synuclein with 1 mM lipid SUVs. SUV composition: DOPC/DOPE/DOPS = 60:25:15. Data were collected at 13 °C on a 700 MHz spectrometer; Figure S8: Fits to DEST and ΔR_2_ data from wild-type alpha-synuclein using different models. (A) DEST profiles of select residues from different regions of the protein, measured at 700 MHz, at 175 and 400 Hz saturation bandwidths (red and blue circles, respectively). The dashed lines represent fits obtained using a two-state model, and the solid lines represent fits obtained using a pseudo-two-state model (see text). (B) Residue-specific ΔR_2_ values (black circles connected by dashed line) with fits obtained using a two-state model (blue circles) or a pseudo-two-state model (red circles). Error bars represent 1 standard deviation; Figure S9: Residue-specific K_3_ equilibrium constants for direct contact and tethered states. K_3_ values were determined by simultaneous fitting of DEST and R_2_ relaxation experiments for alpha-synuclein variants (WT, A30P, 3AE and 4G) that were best fit using a pseudo-two-state model (see text). Error bars represent 1 standard deviation; Figure S10: Schematic for the two-state and pseudo-two-state models used to fit the DEST and ΔR_2_ data. (A) In the two-state model, binding of all residues occurs by conversion of a single molecular free state ensemble, $E_{\mathrm{visible}}^{m}$, to a single molecular bound state ensemble, $E_{\mathrm{dark}}^{m}$, and is dominated by same global $k_{\mathrm{on}}^{\mathrm{app}}$ and $k_{\mathrm{off}}$ rate constants. (B) In the pseudo-two-state model, each residue sampling of a local free-state ensemble, $E_{\mathrm{visible}}^{\mathrm{res}}\left(i\right)$, transitions upon binding to an ensemble of states in which this residue is directly bound to the vesicle surface, $E_{\mathrm{contact}}^{\mathrm{res}}\left(i\right)$, or to an ensemble of states in which this residue is tethered to the surface by nearby directly bound residues, $E_{\mathrm{tethered}}^{\mathrm{res}}\left(i\right)$, via residue-specific on-rate constants $k_{1}^{\mathrm{app}}\left(i\right)$ or $k_{2}^{\mathrm{app}}\left(i\right)$, respectively (see [42,77]); Figure S11: Protein expression levels in RBL-2H3 cells quantified using alpha-synuclein immunostaining under (A) conditions of high expression for the WT and linker mutants and (B) conditions of low and high expression for the 4G mutant. All data were collected using the same staining protocol and microscopy settings. Movies: Representative movies of exocytosis experiments for cells transfected with pcDNA, WT (low expression) or WT (high expression), 3AE (low expression), 3AE (high expression), 4G (low expression) or 4G (high expression) variants of alpha-synuclein.
