# Supplementary material for: The Role of Membrane Affinity and Binding Modes in Alpha-Synuclein Regulation of Vesicle Release and Trafficking
Source: Biomolecules. 2022 Dec 5;12(12):1816. doi: 10.3390/biom12121816 (PMC9775087; doi:10.3390/biom12121816)
Supplement: Supplementary file 1 [file biomolecules-12-01816-s001.zip › Fig S11.pdf]

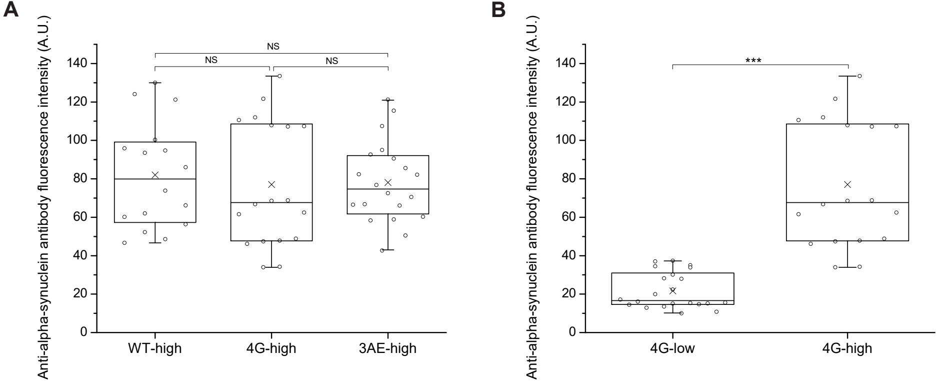

A
B
Anti-alpha-synuclein antibody fluorescence intensity (A.U.)
0
20
40
60
80
100
120
140
NS
NS
NS
***
WT-high
4G-high
3AE-high
4G-low
4G-high

Fig S11
