# Supplementary figures and images for: The Role of Membrane Affinity and Binding Modes in Alpha-Synuclein Regulation of Vesicle Release and Trafficking
Source: Biomolecules. 2022 Dec 5;12(12):1816. doi: 10.3390/biom12121816 (PMC9775087; doi:10.3390/biom12121816)

**A**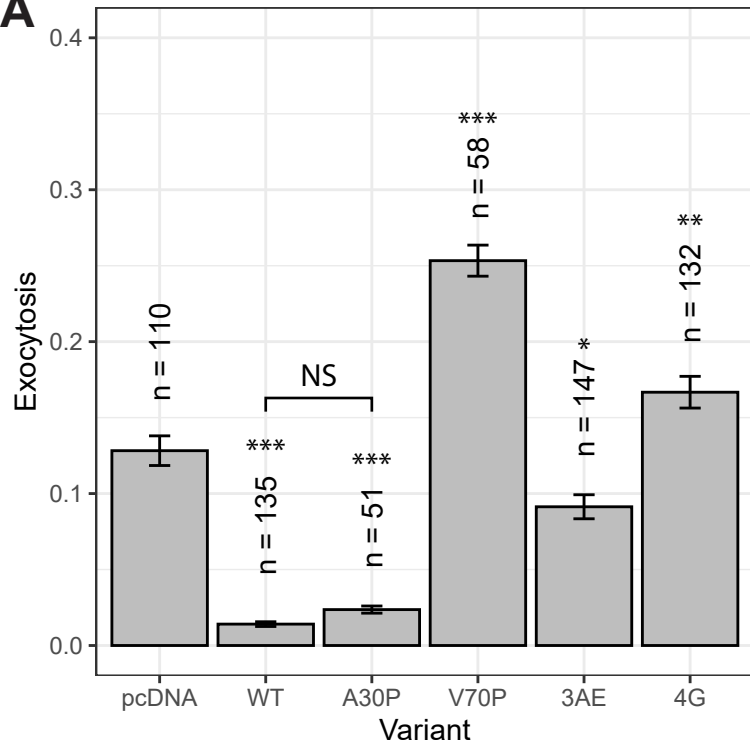**B**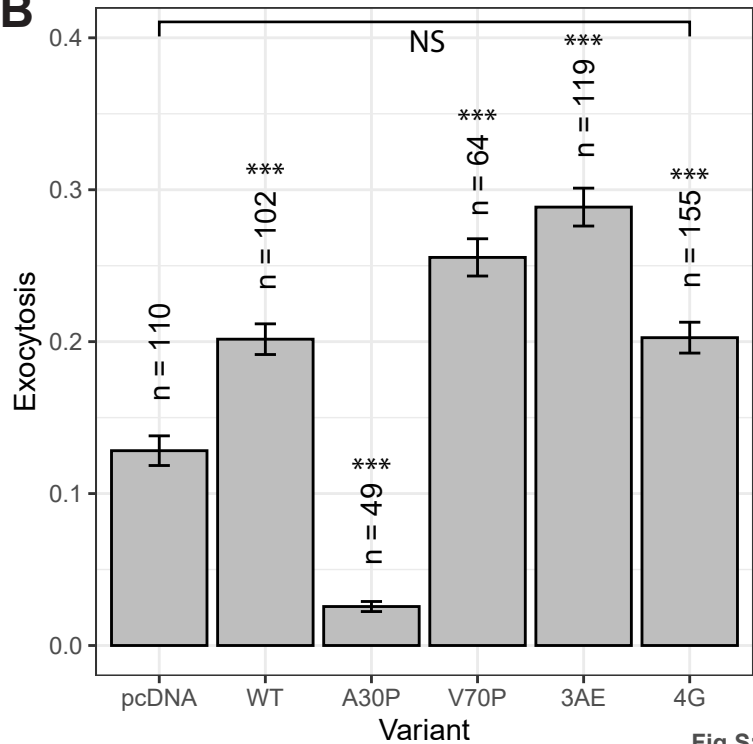**Fig S1**

Supplement: Supplementary file 1 [file biomolecules-12-01816-s001.zip › Fig S1.pdf]

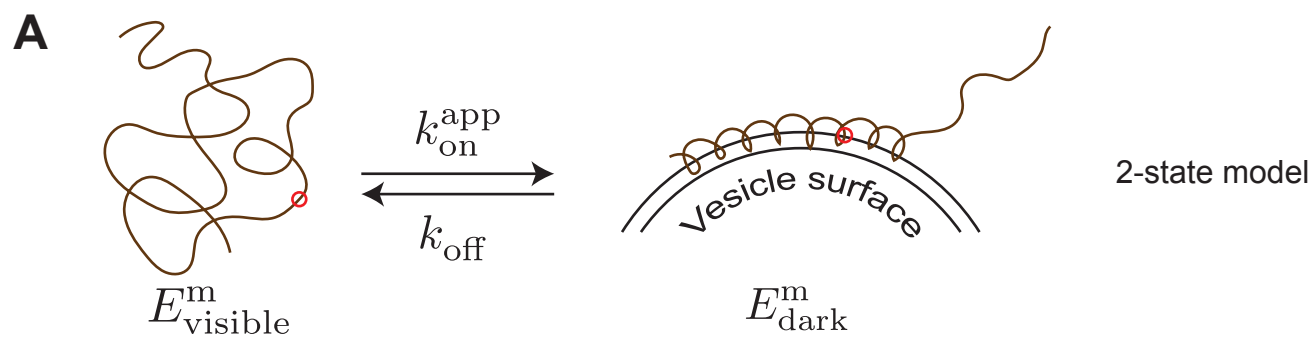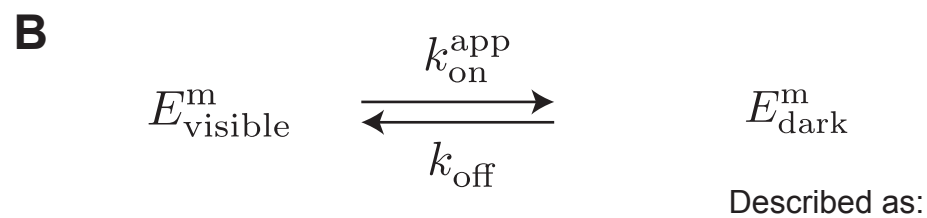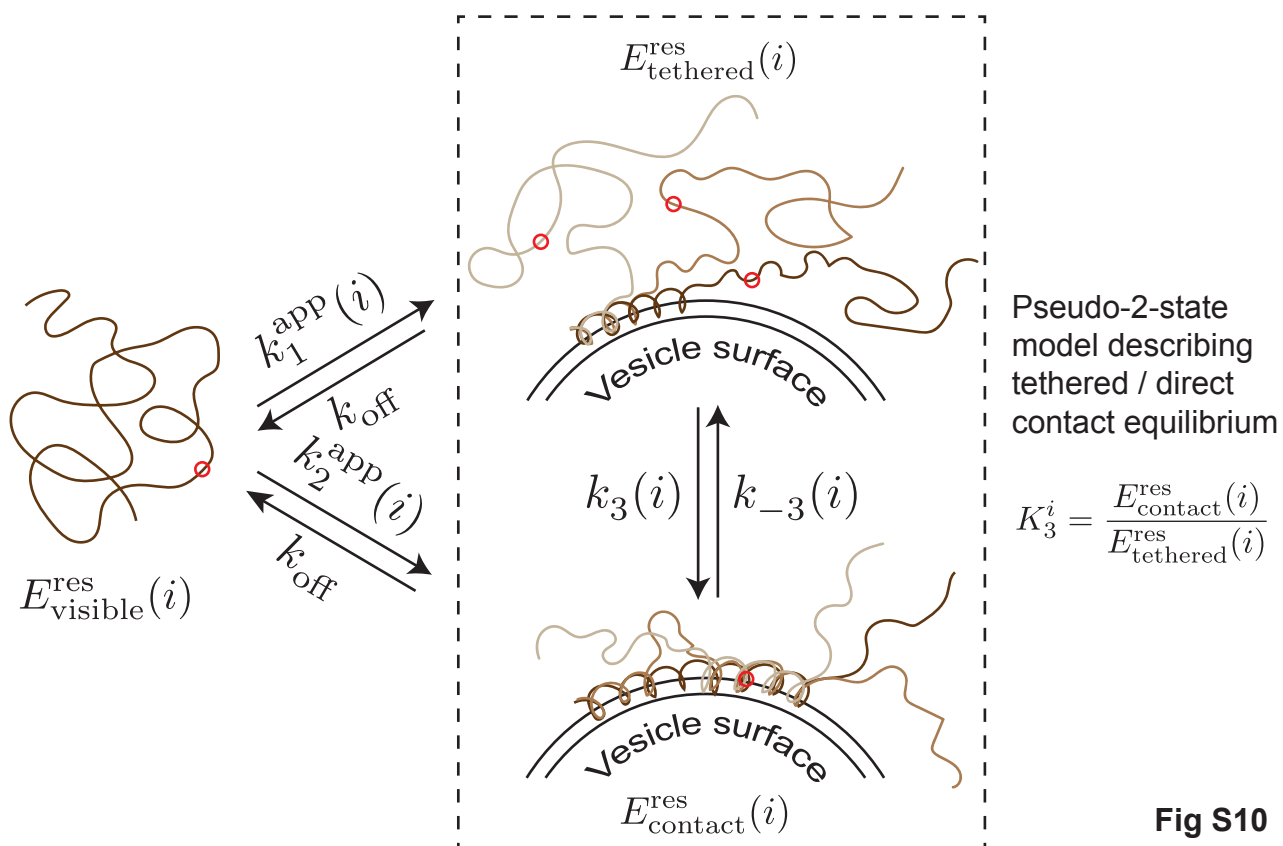

**Fig S10**

Supplement: Supplementary file 1 [file biomolecules-12-01816-s001.zip › Fig S10.pdf]

**A**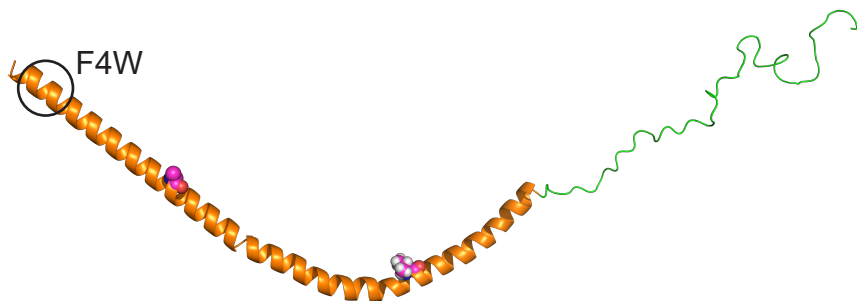**B**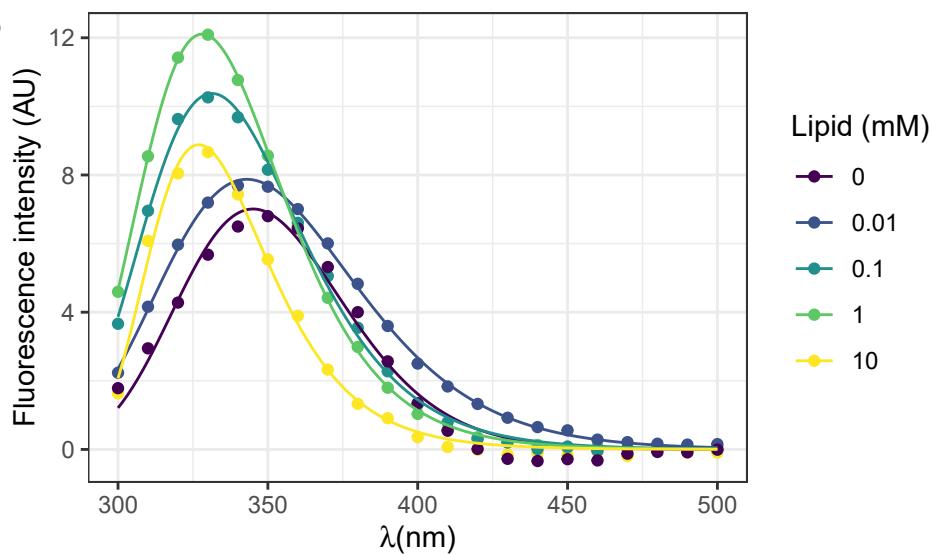**C**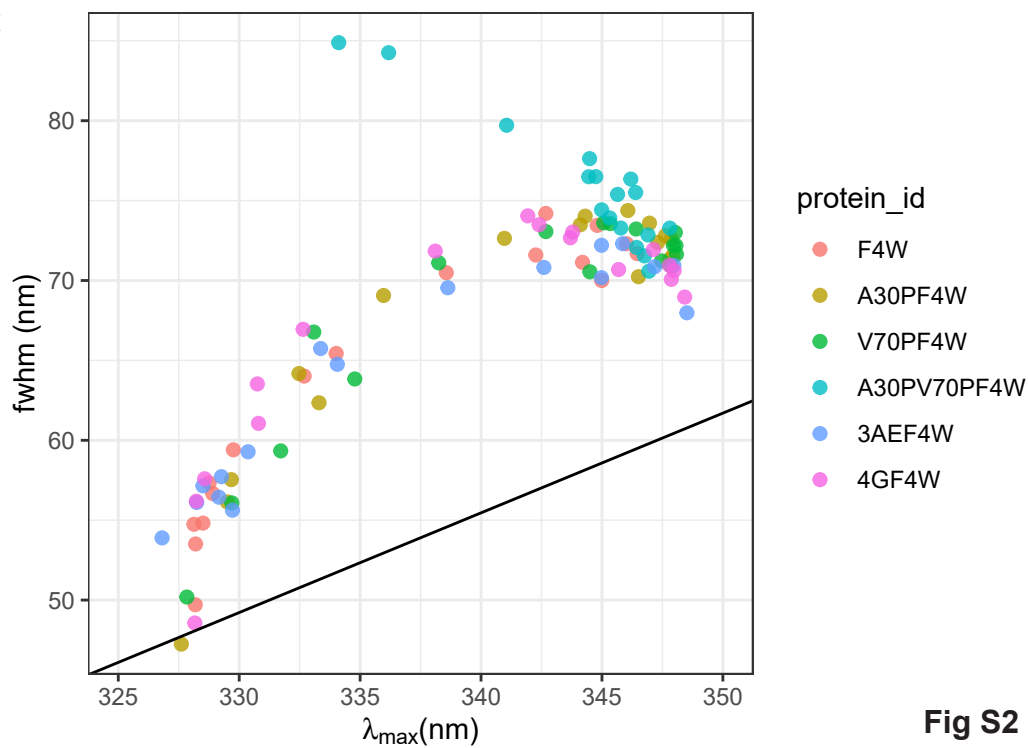**Fig S2**

Supplement: Supplementary file 1 [file biomolecules-12-01816-s001.zip › Fig S2.pdf]

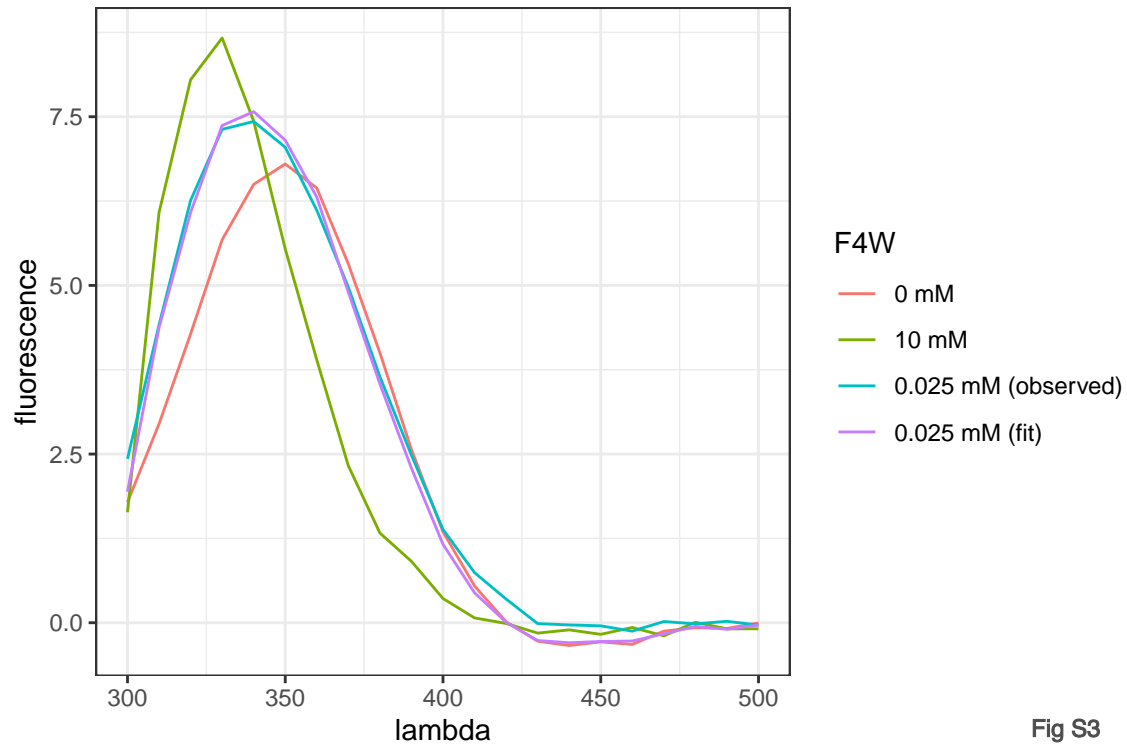

Fig S3

Supplement: Supplementary file 1 [file biomolecules-12-01816-s001.zip › Fig S3.pdf]

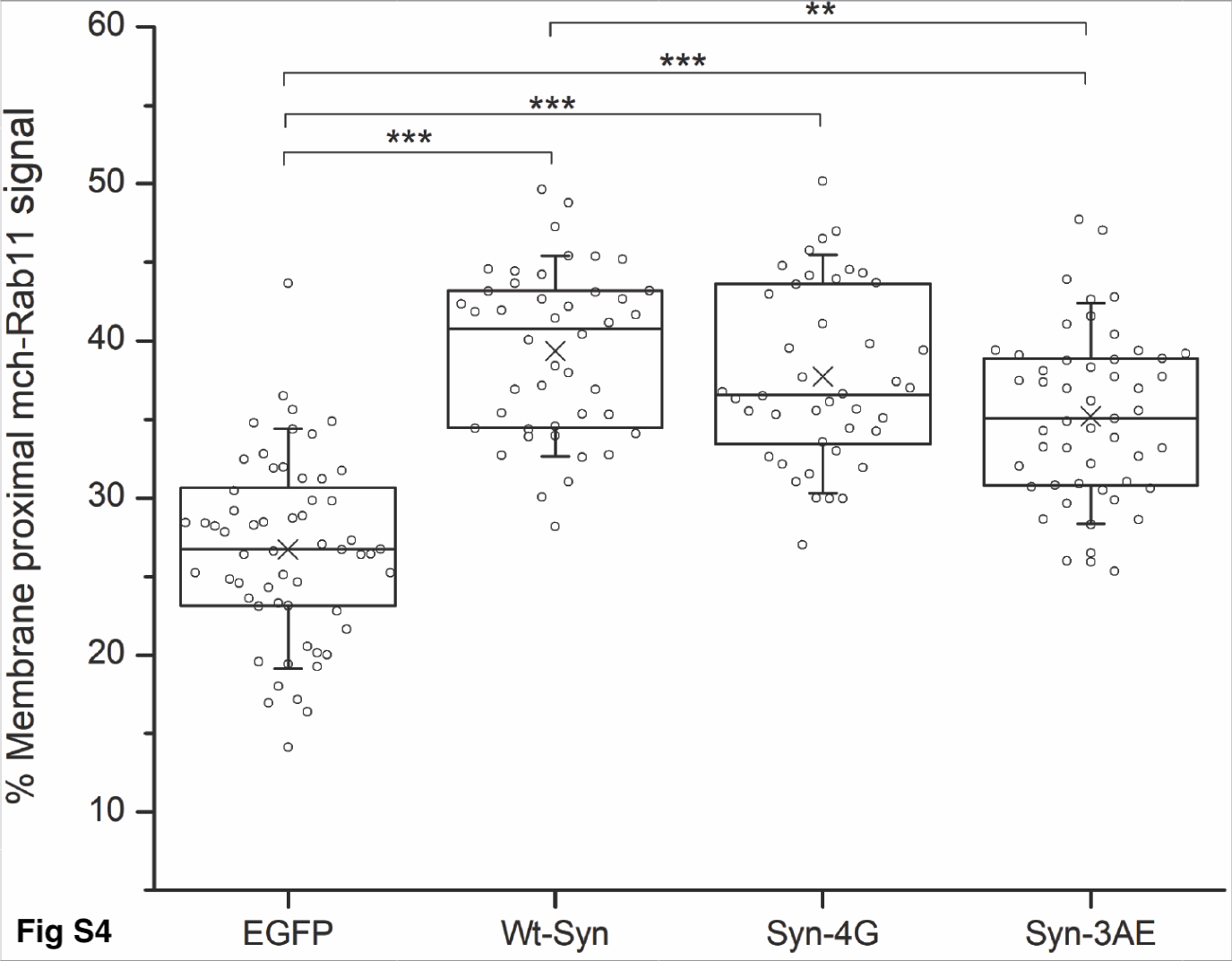

Supplement: Supplementary file 1 [file biomolecules-12-01816-s001.zip › Fig S4.tif]

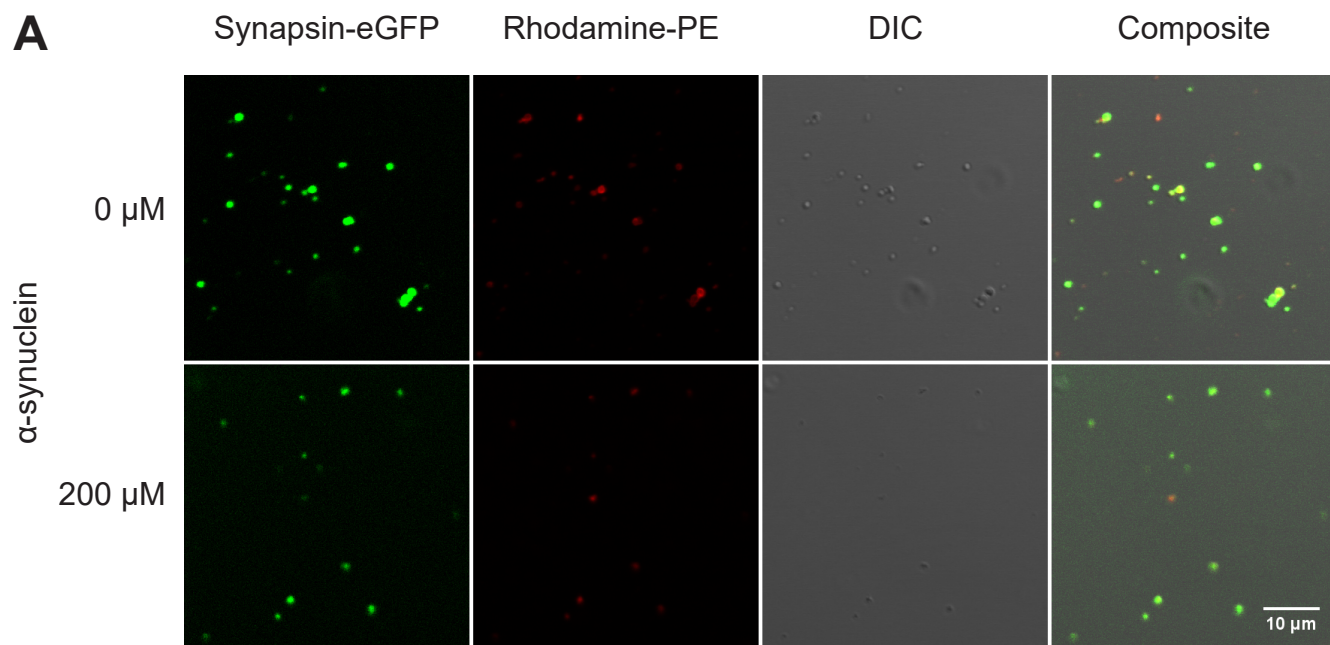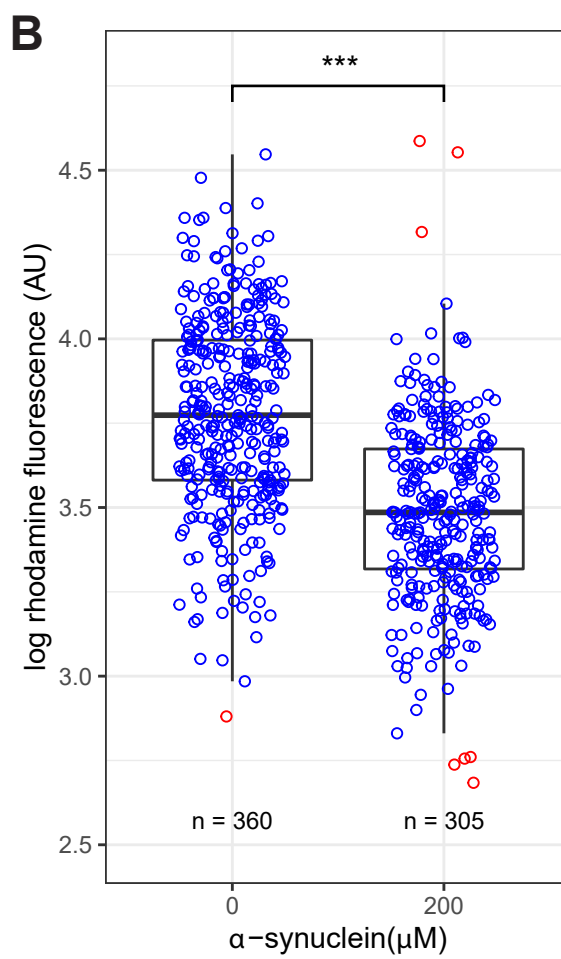

**Fig S5**

Supplement: Supplementary file 1 [file biomolecules-12-01816-s001.zip › Fig S5.pdf]

Peak height ratio

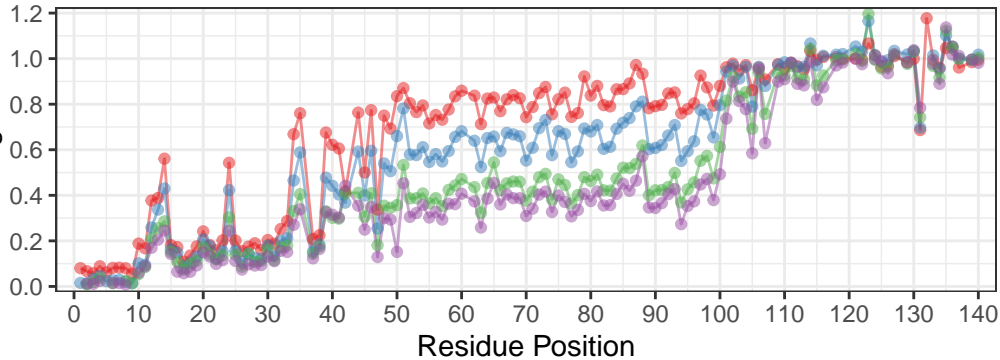

Lipid (mM)

- 2.5
- 5
- 10
- 15

Fig S6

Supplement: Supplementary file 1 [file biomolecules-12-01816-s001.zip › Fig S6.pdf]

**A**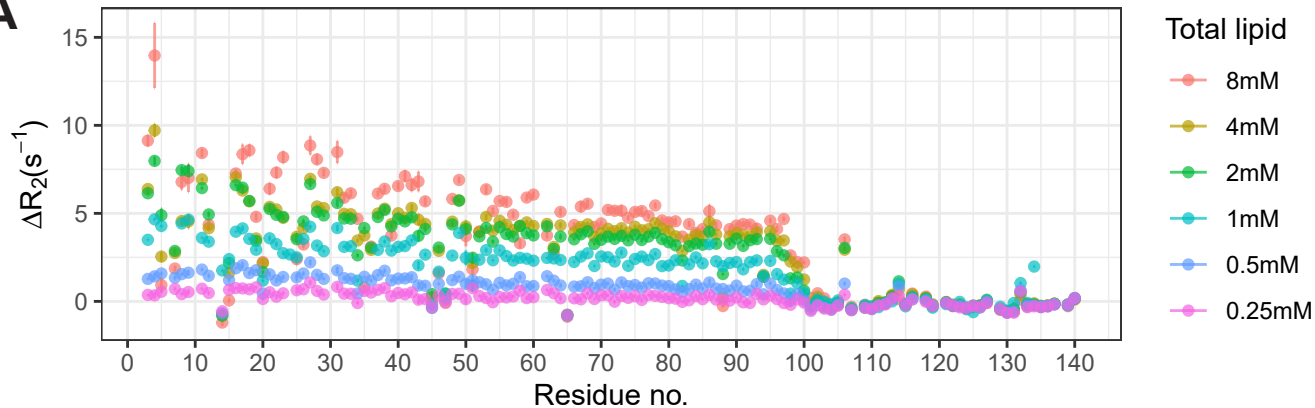**B**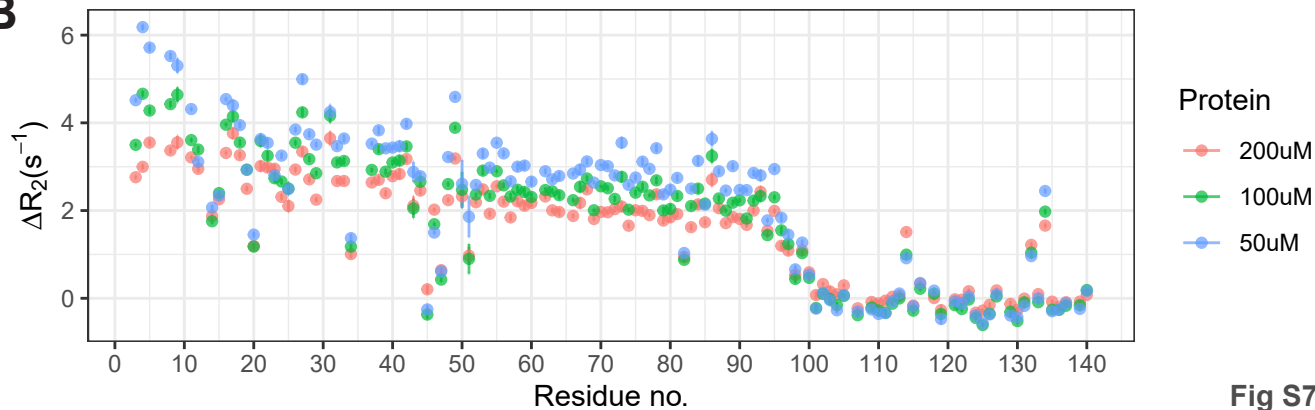**Fig S7**

Supplement: Supplementary file 1 [file biomolecules-12-01816-s001.zip › Fig S7.pdf]

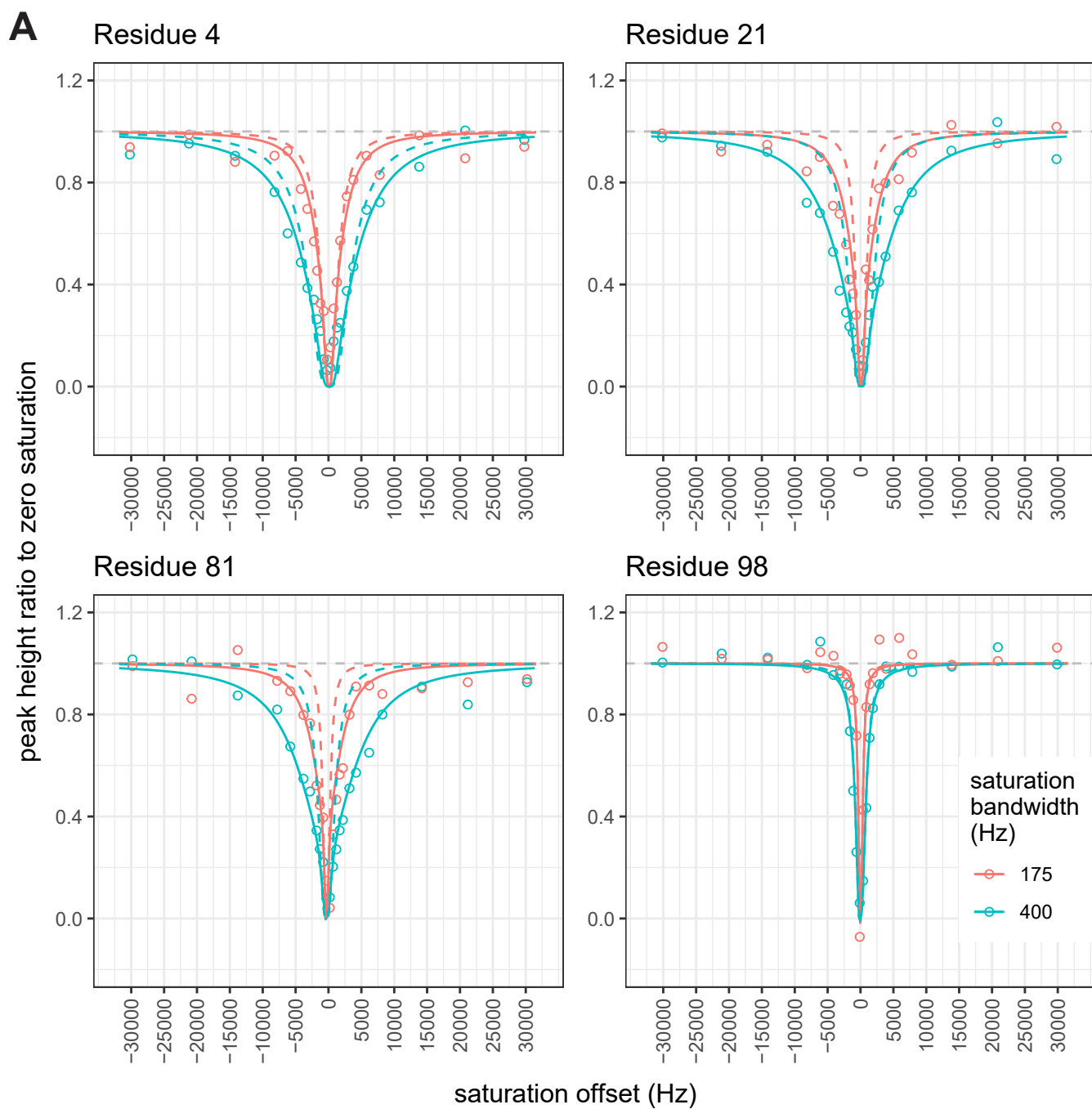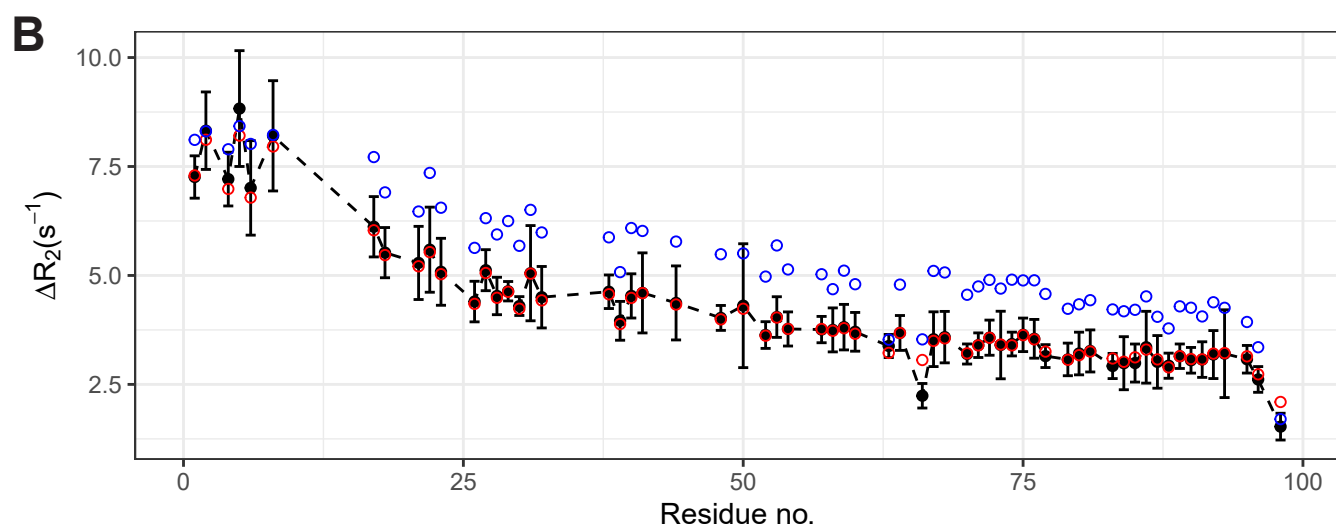

Fig S8

Supplement: Supplementary file 1 [file biomolecules-12-01816-s001.zip › Fig S8.pdf]

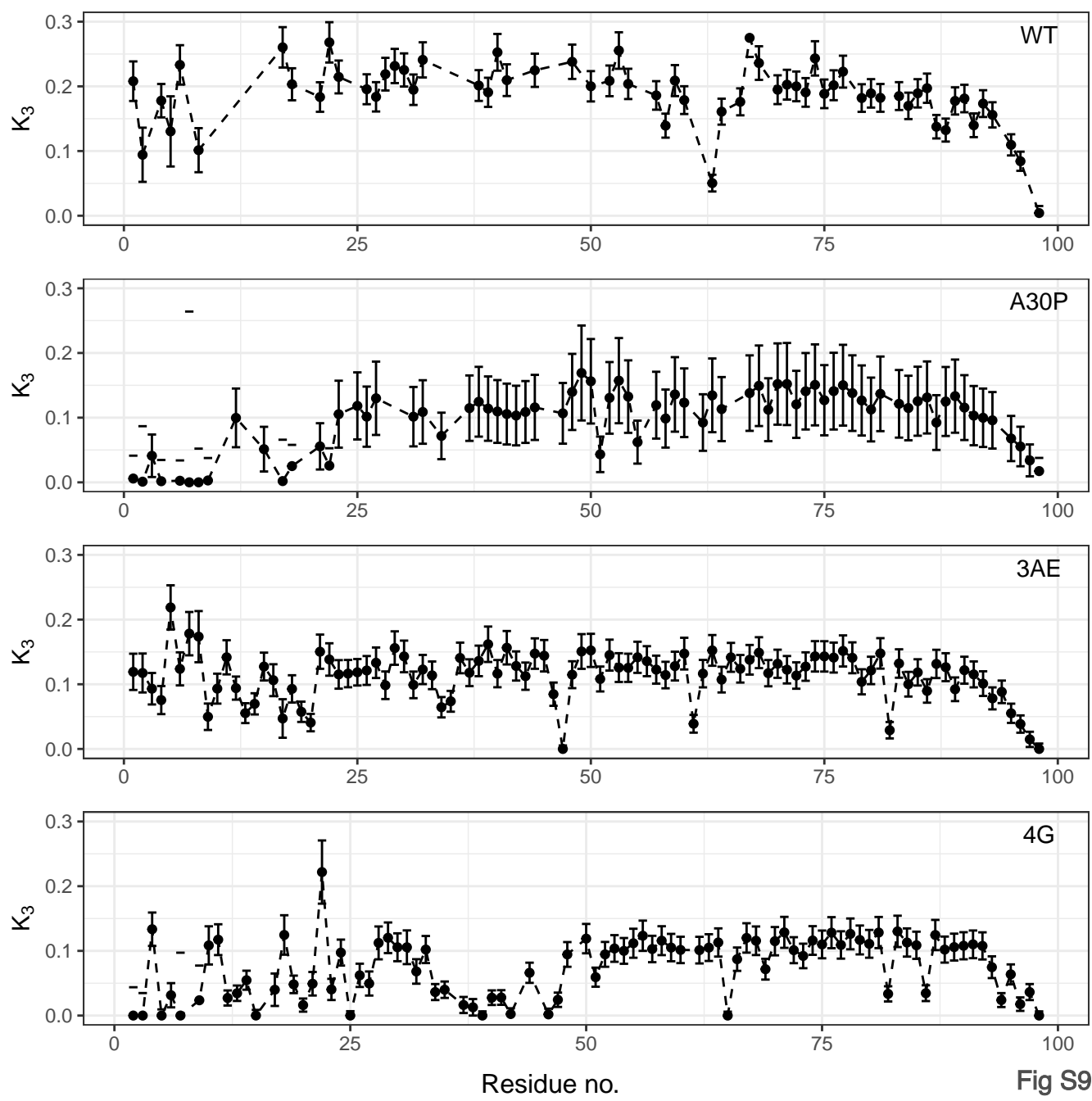

Fig S9

Supplement: Supplementary file 1 [file biomolecules-12-01816-s001.zip › Fig S9.pdf]
